# Gene Expression Profile Induced by Two Different Variants of Street Rabies Virus in Mice

**DOI:** 10.3390/v14040692

**Published:** 2022-03-27

**Authors:** Camila M. Appolinário, Janet M. Daly, Richard D. Emes, Fabio Albuquerque Marchi, Bruna Leticia Devidé Ribeiro, Jane Megid

**Affiliations:** 1 Faculdade de Medicina Veterinária e Zootecnia, Universidade Estadual Paulista, Julio de Mesquita Filho, Distrito de Rubião Júnior, s/n, CEP, Botucatu 18618-970, SP, Brazil; brunadevide@yahoo.com.br; 2 School of Veterinary Medicine and Science, University of Nottingham, Sutton Bonington Campus, Loughborough LE12 5RD, UK; janet.daly@nottingham.ac.uk (J.M.D.); richard.emes@nottingham.ac.uk (R.D.E.); 3 International Research Center, A.C Camargo Cancer Center, São Paulo 01509-900, SP, Brazil; fabio.marchi@accmargo.org.br

**Keywords:** rabies virus, dog, vampire bat, mice, microarray analysis, pathogenesis

## Abstract

Pathogenicity and pathology of rabies virus (RABV) varies according to the variant, but the mechanisms are not completely known. In this study, gene expression profile in brains of mice experimentally infected with RABV isolated from a human case of dog rabies (V2) or vampire bat-acquired rabies (V3) were analyzed. In total, 138 array probes associated with 120 genes were expressed differentially between mice inoculated with V2 and sham-inoculated control mice at day 10 post-inoculation. A single probe corresponding to an unannotated gene was identified in V3 versus control mice. Gene ontology (GO) analysis revealed that all of the genes upregulated in mice inoculated with V2 RABV were involved in the biological process of immune defense against pathogens. Although both variants are considered pathogenic, inoculation by the same conditions generated different gene expression results, which is likely due to differences in pathogenesis between the dog and bat RABV variants. This study demonstrated the global gene expression in experimental infection due to V3 wild-type RABV, from the vampire bat *Desmodus rotundus*, an important source of infection for humans, domestic animals and wildlife in Latin America.

## 1. Introduction

Rabies is a zoonotic, highly lethal and neglected disease that has been affecting humanity for more than 4000 years. Lyssaviruses, such as rabies virus (RABV) enter the body through wounds or by direct contact with mucosal surfaces. The number of human deaths globally due to dog-mediated rabies is estimated to be in excess of 59,000 cases annually with most deaths (around 98%) occurring in Asia and Africa [1]. In contrast, in some countries of the Americas where canine rabies has been eliminated, bats are responsible for the majority of human cases [2,3].

Invasion of the central nervous system (CNS) by RABV occurs by binding to various neuronal receptors, including acetylcholine, the neuronal cell adhesion molecule, or the neuronal growth factor receptor [4]. Viral replication occurs in the neuronal cell body, reached by retrograde axonal transport. Some bat RABV variants may propagate via sensory nerves due to skin tropism [5]. Although clinical signs of rabies are severe, there is minimal inflammation and neuronal destruction compared to other encephalitic viruses [4,6,7]. Variants of RABV differ in the pathogenicity [4,7], but the mechanisms are not completely known. In a detailed analysis of clinical features in published human cases of dog- (V2) or bat-acquired (V3) rabies, Udow et al. (2013) reported that encephalopathy, hydrophobia and aerophobia were more common in dog-acquired rabies, whereas myoclonus, cranial nerve abnormalities and motor and sensory abnormalities were more common in bat-acquired rabies [8]. 

In a previous study [9], we demonstrated that cytokine and chemokine genes associated with the immune response, measured by reverse transcription and quantitative PCR, were differentially expressed in the CNS of mice inoculated with canine or bat RABV variants. Here, we extend these studies to a microarray analysis of brain tissue from mice inoculated with the same variants at 5- and 10-days post inoculation (d.p.i.), to evaluate the gene expression induced by these viruses. 

## 2. Materials and Methods

### 2.1. Virus Strains

Two wild-type RABV variants, isolated from human patients infected by a dog variant (V2) or a vampire bat variant (V3) were used to inoculate mice. Both viruses were obtained at the fourth intracerebral passage in mice.

### 2.2. Experimental Design

Specific pathogen free female C57/BL6 mice, 4 to 6 weeks old, were provided by Cemib [University of Campinas (UNICAMP) animal facility]. The animals were kept in ventilated cabinets with HEPA (High Efficiency Particulate Air) filters and fed irradiated food and sterile water “ad libitum”. In this experiment a total of 64 animals were divided into eight groups: three groups for V2, three groups for V3, and two groups left non-infected. Animals were inoculated, intramuscularly (i.m.) in the right hind limb with 100 µL of viral inoculum with a dose of 40 LD_50_ (50% lethal dose). For both viruses, the LD_50_ was determined by intracranial inoculation of mice. Control groups (*n* = 16), named as non-inoculated, received only viral diluent via i.m., and inoculated groups (*n* = 48) were inoculated i.m. with viral inoculum, V2 or V3. For each viral variant, one of these groups was maintained over a 30-day period, named as the 30-day period evaluation group, and mice in the other two groups were euthanized at either 5 or 10 d.p.i. when whole brain tissue was collected. Non-inoculated animals were killed as well, at 5 or 10 d.p.i. to serve as controls for the microarray comparison (Table 1). Animals in all groups were weighed and evaluated daily for the onset of clinical signs of infection such as ruffled fur, hunched back, hypo-/hyper-excitability, paralysis of one/both hind limbs and tetraplegia. In the 30-day period evaluation group, animals were killed when they reached a semi-comatose state, which was determined as the humane endpoint. Animals were killed by isoflurane inhalation. 

### 2.3. RNA Extraction and RT-qPCR

Brain tissue RNA was extracted with the Invitek® kit (Berlin, Germany) according to the manufacturer’s instructions and stored at −80 °C. Before microarray analysis was performed, the presence of RABV in the brain was confirmed by detection of the RABV N protein gene by RT-qPCR as previously described [9]. Validation of the results obtained in the microarray were made by RT-qPCR using Mouse Quantitect^®^ Primer Assay (Qiagen^®^, Hilden, Germany) for specific cytokines and chemokines as previously described [9].

### 2.4. Microarray

Whole brain was homogenized, and total RNA was isolated from brain samples collected at 5 and 10 d.p.i. from infected animals and controls using Trizol (Invitrogen®, Carlsbad, CA, USA) and purified with the RNeasy Mini-kit and RNase Free DNase Set (Qiagen^®^, Hilden, Germany). Total RNA was quantified by absorbance at 260 nm. The quality of all RNA preparations was controlled by electrophoresis on agarose gels, and they were also evaluated on an Agilent Bioanalyser instrument (Agilent Technologies, Palo Alto, CA, USA). Only samples with a preserved rRNA ratio (28S/18S), no evidence of RNA degradation, and RNA integrity values ≥8 at the Bioanalyser were used in the microarray hybridization and qPCR. 

For the microarray analysis, 1 µg of total RNA was labeled with the Cy3 and Cy5 fluorophores array kit (Affymetrix, Santa Clara, CA, USA). The GeneChip^®^ Mouse Gene 2.0 ST Array, a universal arrangement of Affymetrix (Affymetrix, Santa Clara, CA, USA) that interrogates 35,420 genes was used. Hybridization signals were quantified using a ScanArray Express (PerkinElmer, Boston, MA, USA) and the images were processed using GenePix version 4.0 (Axon, Union City, CA, USA). The CEL files were processed and normalized with the RMA program, after this step an ANOVA analysis was performed between the groups using the LIMMA package (version 3.14) Differentially expressed genes were identified in infected mice compared to the control group for each RABV variant (FDR corrected *p* value < 0.05 log2 difference of expression of >|1|). Annotation of genes enriched in specific functions were determined with reference to the Kyoto Encyclopedia of Genes and Genomes (KEGG) and gene ontology (GO), using “NIPA” software (https://github.com/ADAC-UoN/NIPA (accessed on 20 March 2022); version 0.6.7). Raw data and experimental design are available from ArrayExpress (https://www.ebi.ac.uk/arrayexpress/ (accessed on 20 March 2022) under the accession E-MTAB-11414).

### 2.5. Statistical Analysis

Kaplan–Meier survival curves were plotted and Log–Rank (Mantel–Cox), Grehan–Breslow–Wilcoxon and Mann–Whitney tests were performed in GraphPad Prism version 8.1.2 for Windows (GraphPad, La Jolla, CA, USA).

## 3. Results

### 3.1. Assessment of Lethality of RABV Variants V2 and V3

**Figure 1:**
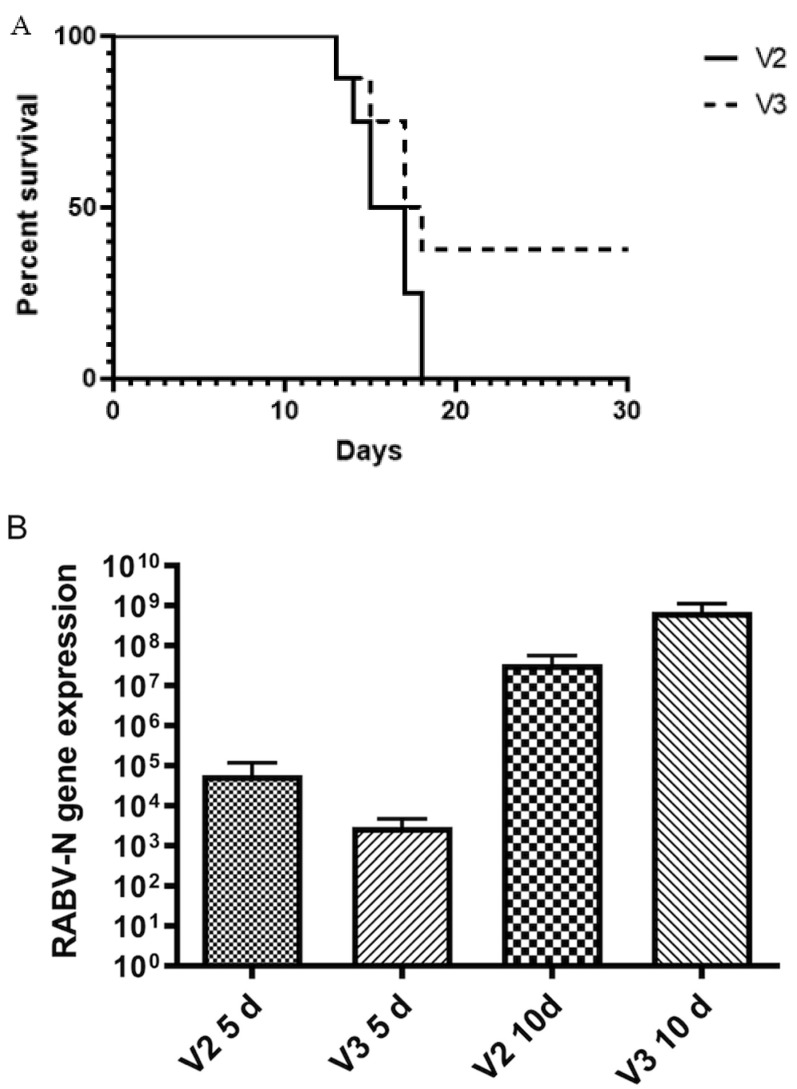
(**A**) Kaplan–Meier survival curve of mice infected with rabies virus (RABV) V2 (canine) and V3 (bat) with no statistic difference between groups. (**B**) *RABV N* gene expression mean in the brains of mice infected with 40 LD_50_ V2 and 40 LD_50_ V3. Mann–Whitney test was applied to compare the results between the groups at 5 and 10 d.p.i. There was no significant difference between the groups at either time point.

Lethality of the viruses was determined by group monitored over a 30-day period post-infection. The incubation period was 12 days and the morbidity period (the time from onset of clinical signs of infection to death) was 5 days for each variant. There was no significant difference in survival nor in the amount of virus in the CNS comparing V2 and V3. Median survival was 16 days for the V2 group and 17.5 days for the V3 group. However, three mice survived with no clinical signs for 30 days in the V3 group (62.5% lethality), whereas all mice were dead at 18 days in the V2 group (lethality 100%) (Figure 1). Animal weights during the 30-day observation period are available in Supplementary File S1.

### 3.2. Transcriptome Analysis of Mouse Brain RNA

To compare the CNS host response to the different RABV variants, whole transcriptome analysis was performed on brain tissue collected from individual mice from all groups at 5 and 10 d.p.i. All animals inoculated with V2 or V3 were positive for the presence of RABV in the brain. 

**Figure 2:**
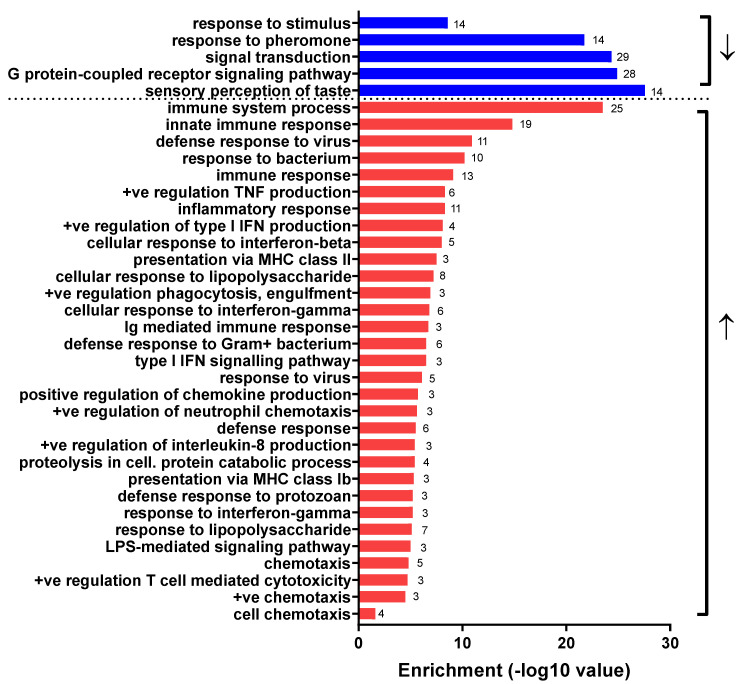
Significantly enriched gene ontology (GO) terms for genes that are down-regulated(blue bar/↓=down regulated) or up-regulated (red bar/↑=up regulated) in brain tissue from mice inoculated with variant V2 compared to control mice at 10 d.p.i. Bars are labelled with gene count. (+ve = positive; Dashed line = visual division between down and up-regulated genes).

Analysis of mice inoculated with V2 or V3 compared to their respective controls at 5 d.p.i. showed no different expressed genes (DEGs). In the V3 group at 10 d.p.i., a single array probe for an unannotated gene was differentially expressed compared with the control mice. In contrast, 138 array probes associated with 120 DEGs were detected between mice inoculated with V2 and the control mice at the same time point (10 d.p.i.). Normalized expression of DEGs within the groups are presented in Supplementary Data (Files S9 and S10). The gene ontology (GO) analysis revealed that all the up-regulated genes were involved in the biological process of immune defense against pathogens (Figure 2). 

The most enriched GO biological process term in the up-regulated genes was GO:0002376 (immune system process) with 25 genes in the differentially expressed gene list. The KEGG pathway analysis highlighted that a large number of genes that were up-regulated after infection with V2 were the same as those upregulated in response to *Herpes simplex* infection (genes *Myd88, Irf7, H2-Q7, Casp8, Tlr3, Daxx, C3, H2-Aa, Oas2, Oas3, H2-T22, H2-T23* and *Cd74*). No enriched KEGG pathways were observed in the down-regulated genes. However, vomeronasal receptor (Vmnr) genes were significantly enriched in each of the GO biological terms of ‘signal transduction’, ‘G protein-coupled receptor signaling pathway’, ‘response to stimulus’, ‘response to pheromone’ and ‘sensory perception of taste’ representing the down-regulated genes. More detail of GO enrichment and KEGG pathway analysis is presented in Supplementary Files S2–S8.

### 3.3. Microarray Results Validation

**Figure 3:**
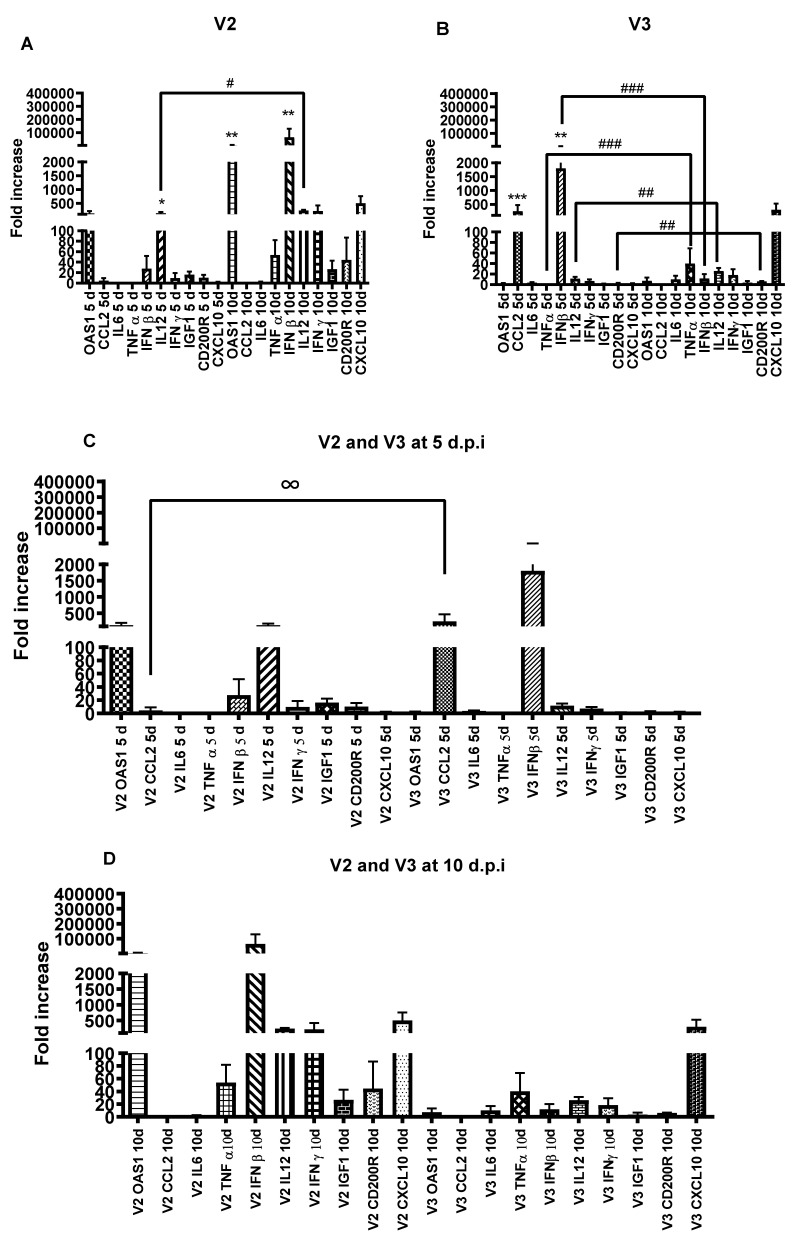
Relative gene expression of cytokines and chemokines in both groups (V2 and V3) at 5 d.p.i and 10 d.p.i. Kruskal–Wallis test was applied to analyze the global results and Mann–Whitney two-tailed test was applied to make individual comparisons at 5 vs. 10 d.p.i. (**A**) Expression at 5 and 10 d.p.i. in the V2 group. * *p* < 0.05; ** *p* = 0.01; # *p* = 0.09. (**B**) Expression at 5 and 10 d.p.i. in the V3 group. ** *p* < 0.01; *** *p* < 0.001; ### *p* = 0.0005; ## *p* = 0.0047. (**C**) Expression at 5 d.p.i. in V2 and V3. ^∞^ *p* < 0.01. (**D**) Expression at 10 d.p.i. in V2 and V3.

To validate the results obtained in the microarray, 11 genes were chosen as important markers of the immune response, namely: *CCL2, OAS1, IL2, IL6, IL12, TNFα, IFN γ, IFNβ, CXCL10, CD200R* and *IGF1* genes (Figure 3). In the V2 group, expression levels of IL-12 were significantly higher (*p* < 0.05) at 5 d.p.i. and at 10 d.p.i., *OAS1* and interferon beta (*IFN-β*) were significantly higher (*p* = 0.01). Comparing 5 d.p.i. vs. 10 d.p.i., IL-12 showed a slight but not significant increase; *IFN-β* (** *p* < 0.01) and *CCL2* (*** *p* < 0.001) had a higher expression at 5 d.p.i. In the V3 group, no difference was observed at 10 d.p.i., although in the comparison between 5 d.p.i. vs. 10 d.p.i. *TNF-α* (*p* = 0.0005), IFN-β (*p* = 0.0002), *IL-12* (*p* = 0.0047) and *CD200R* (*p* = 0.0047) showed a higher expression at 10 d.p.i. 

## 4. Discussion

The V2 RABV variant was more lethal thanV3, in agreement with previous studies [9,10,11,12]. Fuoco et al. (2019) showed that intradermal inoculation of mice with a RABV isolate from an insectivorous bat led to 33% mortality whereas an isolate from a wild canid (*Cerdocyon thous*, crab-eating fox) led to 67% mortality [13].

In our work, the majority of the up-regulated 120 DEGs have their functions associated with cell adhesion, receptor signaling, peptide antigen binding, double-stranded RNA binding and pathways involved in innate immune responses, cellular immune responses, and response to viruses and bacteria. This is in agreement with similar studies in which mice were infected with RABV. Ubol et al. (2006) inoculated 1-day-old mice by the intracerebral route with a primary RABV isolate (passaged once in mouse brain) from an infected dog [14]. They observed altered gene expression at days 2 (29 genes), 4 (109 genes) and 6 (98 genes) post-inoculation in eight major groups: immune response, metabolism, receptor and transport, growth factors, death-mediated factors, transcription and translation factors, proteases and kinases. The up-regulation of *VCAM-1* in the present study demonstrates the participation of this molecule in viral penetration, in which the activation occurred immediately after entry of the virus in the brain and in the absence of clinical signs, corroborating previous works published by our group and others [9,15,16,17].

Results also demonstrate toll-like receptor-3 (*TLR3*) activation associated with upregulation of various immune response genes, innate immune responses, upregulated virus responses indicating chemotaxis, inflammatory and antiviral responses activated by *TLR3* to CNS RABV infection [18]. The early activation of the defense response was only observed in V2, similar to the results obtained by Zhang et al. (2016), who observed activation of the early innate and adaptive immune response induced by infection by the Flury-Hep RABV (an attenuated strain) as opposed to CVS -11 (highly pathogenic strain) [17]. In work by Sugiura et al. (2011), animals inoculated with a lethal dose of CVS intramuscularly did not show any change in gene expression before the onset of clinical signs [19]. This demonstrates that street and fixed virus strains of RABV have distinct expression profiles, corroborated by results obtained in the current study. Mere clinical evaluation of mice in experimental rabies is not able to detect subtle alteration in physiological parameters and behavior and more precise evaluation methods are required.

Besides the immune response pathways, there is also activation of genes associated with the surface and components of the cell and cell matrix, outer cytoplasmic membrane, lysosome, phagocytic vesicles and extracellular space, suggesting alteration in CNS structures and integrity leading to dysfunction, which is a major factor associated with RABV-induced death [20,21]. Neuronal dysfunction can be observed in the absence of classical clinical signs of rabies due to the decreased expression of genes associated with transduction, response to stimuli and pheromones, and taste perception, indicating centrifugal dissemination via sensory nerves and the functional alteration of the CNS resulting from viral replication [5].

Another fact was activation of the canonical pathway associated with viral myocarditis in the infection caused by V2 a clinical finding already described in human rabies cases [22,23].

Although there were no obvious clinical signs in the mice at 10 d.p.i., there was already down-regulation of *Vmnr* genes associated with signal transduction, response to stimulation, response to pheromones and taste perception not described in other studies. This may be due to different ages of mice and routes of inoculation used. 

Moreover, previous studies have not directly compared wild RABV isolates of canine or bat origin in mice inoculated by the intramuscular route, better mimicking a natural infection. The microarray analysis also showed *Vmnr* genes expressed differentially compared to all other genes analyzed. The VMN receptors are located in the vomeronasal organ (VNO), a secondary chemosensory system that is enclosed in a cartilaginous capsule on the medial surface of the nasal septum. The VNO has chemosensory neurons responsible for identifying numerous pheromones that activates a cascade of molecules that leads to the expression of socio-sexual behavior with involvement of the limbic system [24,25]. In rabies, the change in limbic system activity, which involves behavioral and emotional changes, has been recognized for millennia, so much so that the word rabies is rooted in the Hindi rhabaze, which means to become violent, as in animals the limbic system is directly influenced by recognition of pheromones [25]. Although the differential expression of so many *Vmnr* genes needs further studies to determine its real influence on animal rabies, it is already known from inoculation of mice with herpes simplex virus via the intranasal route that neurotropic viruses may invade CNS via vomeronasal sensory neurons [26]. In a study of mice inoculated by intranasal instillation with CVS, olfactory receptor cells were selectively infected [27]. The down-regulation of *Vmnr* genes in mice inoculated intramuscularly with V2 suggests that this variant may spread to or from the CNS by the olfactory route. This is further supported by the herpes simplex infection pathway being the most significant KEGG pathway identified in this study, but such results are yet to be confirmed by different assays. 

Although both RABV variants used in this study are considered pathogenic and the same LD_50_ was used to inoculate mice, different lethality between V2 and V3 as well as different gene profiles were observed. There were some apparent differences between DEGs and expression levels of selected cytokines and chemokines determined by RT-qPCR. This may result from the difference in sensitivity between this technique and from individual variations between genes and animals that are more evident in the real-time PCR analysis [10]. In a study of a pathogenic wildtype-silver-haired bat RABV in mice, Wang et al. (2005) had similar results regarding discrepancies between detection of some genes in microarray compared to RT-qPCR [4]. Very low or very high expression levels as well as differences in the probes used in each technique decrease the concordance between those two assays [10]. Another important point that might influence the magnitude of gene expression is the clinical status of animals when sampled. In the current study, none of the animals presented clinical signs when samples were collected at 5 or 10 d.p.i. The same observation was made in a microarray study published by Sugiura et al. (2011) in which mice inoculated intramuscularly with CVS at day 3 showed no illness nor significant changes in signal of brain or spinal cord [19]. 

Despite the discrepancies, the RT-qPCR results confirm the microarray analysis showing that V2 induces expression of a larger number of the analyzed genes and at a higher intensity, especially at 10 d.p.i., compared with the V3 RABV variant. Similarly, in the study by Wang et al. (2005), mice inoculated with the same lethal dose of a highly pathogenic wildtype-silver-haired bat RABV or an attenuated CVS strain (lab strain) via the intracranial or intramuscular routes, showed that the genes upregulated by the attenuated strain were higher in both number and intensity when compared to the pathogenic bat RABV [4]. With the progression of the infectious process, at 10 d.p.i., there was an apparent increase in gene expression of all cytokines and chemokines compared to 5 d.p.i. The increase in cytokines and chemokines during RABV infection, has been reported by others [28,29,30,31] and is associated with viral pathogenicity [32], which is also in agreement with our results, as higher gene expression is associated with higher lethality [28,32].

The gene expressions of cytokines and chemokines in the V2 and V3 groups were also qualitatively distinct, especially at 10 d.p.i. In the V2 group at 5 d.p.i, a higher expression of *IL12* was detected while in the V3 group there was an increase in *IFNβ* and *CCL2*. At 10 d.p.i., in the V2 group there was an increase in OAS1 and IFNβ, while in the V3 group there was no increased cytokine or chemokine expression. In contrast to what was observed for the other cytokines, in the V3 group, *IFNβ* expression was higher at 5 d.p.i compared to 10 d.p.i. after inoculation, when its levels drastically reduced. This same expression profile was observed by Chopy et al. (2011) [16], probably due to the evasion mechanism that interferes with the phosphorylation of *IRF3* and the translocation of *STAT 2* to the nucleus, blocking the production of type 1 *IFN* [16,33]. The increase in *IFNβ* occurred late in V2 group but was not strong enough to diminish viral replication. Levels of *RABV-N* were higher at 10 d.p.i. when compared to 5 d.p.i. (*p* < 0.001). Higher expression of *CCL2* was observed, at 5 d.p.i. in the V3 group, highlighting the possibility of greater permeability of blood–brain barrier (BBB) induced by this chemokine compared to V2. This is in agreement with the lower relative lethality observed for this variant as the increase in BBB permeability has been associated with greater penetration of effector cells [32,34,35], contributing to viral elimination [34,36]. 

In conclusion, our results demonstrate that the microarray study of two wild type RABV, V2 and V3, have a gene expression profile intrinsic to the viral variant. In the V2 group, a significant difference of signal compared to control mice was only seen at 10 d.p.i. The majority of the 120 up-regulated DEGs were associated with multiple functions in agreement to similar published studies. The V3 group showed only one differently expressed gene with non-annotated function at 10 d.p.i.. However, RT-q PCR showed that some important cytokines and chemokines were detected at 5 and 10 d.p.i. in the V3 group. This may partially explain the lower lethality rate in mice inoculated with V3 and the differences in gene expression between both wild RABV isolates. Such research not only provides insights into the basic pathology of lyssaviruses but also may prove important towards future treatment modalities.

## Figures and Tables

**Table 1:** **Experimental design.** A total of 64 specific pathogen free, female, C57/BL6 mice, 4 to 6 weeks old, were used in this study: 24 were inoculated via i.m. with 100 µL of V2 or V3 (40*LD*_50_) and 16 were inoculated with viral diluent (non-infected).

Experimental Procedure	V2 (Number of Animals)	V3 (Number of Animals)	Non-Infected (Number of Animals)
Viral inoculation	Yes (24)	Yes (24)	-
Only viral diluent inoculation	-	-	Yes (16)
30-day period evaluation	Yes (8)	Yes (8)	-
Killed 5 d.p.i. *	Yes (8)	Yes (8)	Yes (8)
Killed 10 d.p.i. *	Yes (8)	Yes (8)	Yes (8)
Daily weights and clinical evaluation	Yes (24)	Yes (24)	Yes (16)

(*) Brains were collected from animals killed at 5- or 10-days post inoculation (d.p.i.) and processed for microarray and RT-qPCR; (-) Absence of the group.

## Supplementary Materials

The following supporting information can be downloaded at: https://www.mdpi.com/article/10.3390/v14040692/s1, File S1: Mean weight of infected mice, Files S2–S7: up- and down-regulated Gene Ontology terms, File S8: KEGG pathways, File S9: Heatmap of normalized expression levels; File S10: Combined significant DEGs.

## References

[1] Hampson K., Coudeville L., Lembo T., Sambo M., Kieffer A., Attlan M., Barrat J., Blanton J.D., Briggs D.J., Cleaveland S. (2015). Estimating the global burden of endemic canine rabies. PLoS Negl. Trop. Dis..

[2] Pieracci E.G., Pearson C.M., Wallace R.M., Blanton J.D., Whitehouse E.R., Ma X., Stauffer K., Chipman R.B., Olson V. (2019). Vital Signs: Trends in Human Rabies Deaths and Exposures—United States, 1938–2018. MMWR Morb. Mortal. Wkly. Rep..

[3] Vigilato M.A., Cosivi O., Knobl T., Clavijo A., Silva H.M. (2013). Rabies update for Latin America and the Caribbean. Emerg. Infect. Dis..

[4] Wang Z.W., Sarmento L., Wang Y., Li X.Q., Dhingra V., Tseggai T., Jiang B., Fu Z.F. (2005). Attenuated rabies virus activates, while pathogenic rabies virus evades, the host innate immune responses in the central nervous system. J. Virol..

[5] Hemachudha T., Ugolini G., Wacharapluesadee S., Sungkarat W., Shuangshoti S., Laothamatas J. (2013). Human rabies: Neuropathogenesis, diagnosis, and management. Lancet Neurol..

[6] Jackson A.C., Randle E., Lawrance G., Rossiter J.P. (2008). Neuronal apoptosis does not play an important role in human rabies encephalitis. J. Neurovirol..

[7] Morimoto K., Hooper D.C., Spitsin S., Koprowski H., Dietzschold B. (1999). Pathogenicity of different rabies virus variants inversely correlates with apoptosis and rabies virus glycoprotein expression in infected primary neuron cultures. J. Virol..

[8] Udow S.J., Marrie R.A., Jackson A.C. (2013). Clinical features of dog- and bat-acquired rabies in humans. Clin. Infect. Dis..

[9] Appolinario C.M., Allendorf S.D., Peres M.G., Ribeiro B.D., Fonseca C.R., Vicente A.F., Antunes J.M., Megid J. (2016). Profile of Cytokines and Chemokines Triggered by Wild-Type Strains of Rabies Virus in Mice. Am. J. Trop. Med. Hyg..

[10] Etienne W., Meyer M.H., Peppers J., Meyer R.A. (2004). Comparison of mRNA gene expression by RT-PCR and DNA microarray. Biotechniques.

[11] Appolinario C., Allendorf S.D., Vicente A.F., Ribeiro B.D., Fonseca C.R., Antunes J.M., Peres M.G., Kotait I., Carrieri M.L., Megid J. (2015). Fluorescent antibody test, quantitative polymerase chain reaction pattern and clinical aspects of rabies virus strains isolated from main reservoirs in Brazil. Braz. J. Infect. Dis. Off. Publ. Braz. Soc. Infect. Dis..

[12] Appolinario C.M., Allendorf S.D., Peres M.G., Fonseca C.R., Vicente A.F., Antunes J.M.A.d.P., Pantoja J.C.F., Megid J. (2015). Evaluation of short-interfering RNAs treatment in experimental rabies due to wild-type virus. Braz. J. Infect. Dis..

[13] Fuoco N.L., Fernandes E.R., Guedes F., Dos Ramos Silva S., Guimaraes L.P., Silva N.U., Ribeiro O.G., Katz I.S.S. (2019). *Nyctinomops laticaudatus* bat-associated Rabies virus causes disease with a shorter clinical period and has lower pathogenic potential than strains isolated from wild canids. Arch. Virol..

[14] Ubol S., Kasisith J., Mitmoonpitak C., Pitidhamabhorn D. (2006). Screening of upregulated genes in suckling mouse central nervous system during the disease stage of rabies virus infection. Microbiol. Immunol..

[15] Thoulouze M.I., Lafage M., Schachner M., Hartmann U., Cremer H., Lafon M. (1998). The neural cell adhesion molecule is a receptor for rabies virus. J. Virol..

[16] Chopy D., Detje C.N., Lafage M., Kalinke U., Lafon M. (2011). The type I interferon response bridles rabies virus infection and reduces pathogenicity. J. Neurovirol..

[17] Zhang D., He F., Bi S., Guo H., Zhang B., Wu F., Liang J., Yang Y., Tian Q., Ju C. (2016). Genome-Wide Transcriptional Profiling Reveals Two Distinct Outcomes in Central Nervous System Infections of Rabies Virus. Front. Microbiol..

[18] Lafon M., Mégret F., Meuth S.G., Simon O., Velandia Romero M.L., Lafage M., Chen L., Alexopoulou L., Flavell R.A., Prehaud C. (2008). Detrimental contribution of the immuno-inhibitor B7-H1 to Rabies Virus encephalitis. J. Immunol..

[19] Sugiura N., Uda A., Inoue S., Kojima D., Hamamoto N., Kaku Y., Okutani A., Park C., Yamada A. (2011). Gene expression analysis of host immune response in the central nervous system following lethal CVS-11 infection in mice. Jpn J. Infect. Dis..

[20] Li X.Q., Sarmento L., Fu Z.F. (2005). Degeneration of neuronal processes after infection with pathogenic, but not attenuated, rabies viruses. J. Virol..

[21] Feige L., Zaeck L.M., Sehl-Ewert J., Finke S., Bourhy H. (2021). Innate Immune Signaling and Role of Glial Cells in Herpes Simplex Virus- and Rabies Virus-Induced Encephalitis. Viruses.

[22] Raman G.V., Prosser A., Spreadbury P.L., Cockcroft P.M., Okubadejo O.A. (1988). Rabies presenting with myocarditis and encephalitis. J. Infect..

[23] Hofman P., Bourhy H., Michiels J.F., Dellamonica P., Sureau P., Boissy C., Loubière R. (1992). Rabies encephalomyelitis with myocarditis and pancreatitis. Report on a case recently imported into France. Ann. Pathol..

[24] Silva L., Antunes A. (2017). Vomeronasal Receptors in Vertebrates and the Evolution of Pheromone Detection. Annu. Rev. Anim. Biosci..

[25] Awasthi M., Parmar H., Patankar T., Castillo M. (2001). Imaging Findings in Rabies Encephalitis. Am. J. Neuroradiol..

[26] Mori I., Goshima F., Ito H., Koide N., Yoshida T., Yokochi T., Kimura Y., Nishiyama Y. (2005). The vomeronasal chemosensory system as a route of neuroinvasion by herpes simplex virus. Virology.

[27] Lafay F., Coulon P., Astic L., Saucier D., Riche D., Holley A., Flamand A. (1991). Spread of the CVS strain of rabies virus and of the avirulent mutant AvO1 along the olfactory pathways of the mouse after intranasal inoculation. Virology.

[28] Chopy D., Pothlichet J., Lafage M., Megret F., Fiette L., Si-Tahar M., Lafon M. (2011). Ambivalent role of innate immune response in rabies virus pathogenesis. J. Virol..

[29] Saha A., Rangarajan P. (2003). Common host genes are activated in mouse brain by Japanese encephalitis and rabies virus. J. Gen. Virol..

[30] Phares T.W., Kean R.B., Mikheeva T., Hooper D.C. (2006). Regional differenced in blood-brain barrier permeability changes and inflammation in the apathogenic clearance of virus from the central nervous system. J. Immunol..

[31] Solanki A., Radotra B.D., Vasishta R.K. (2009). Correlation of cytokine expression with rabies virus distribution in rabies encephalitis. J. Neuroimmunol..

[32] Zhao L., Toriumi H., Kuang Y., Chen H., Fu Z.F. (2009). The roles of chemokines in rabies virus infection: Overexpression may not always be beneficial. J. Virol..

[33] Phehaud C., Megret F., Lafage M., Lafon M. (2005). Virus infection switches TLR-3 positive human neurons into high producers of interferon-beta. J. Virol..

[34] Kuang Y., Lackay S.N., Zhao L., Fu Z.F. (2009). Role of chemokines in the enhancement of BBB permeability and inflammatory infiltration after rabies virus infection. Virus Res..

[35] Matasani T., Ito N., Shimizu K., Ito Y., Nakagawa K., Sawaki Y., Koyama H., Sugiyama M. (2010). Rabies virus nucleoprotein functions to evade activation of the RIG-I-mediated antiviral response. J. Virol..

[36] Hooper D.C., Roy A., Barkhouse D.A., Li J., Kean R.B. (2011). Rabies virus clearance from the central nervous system. Adv. Virus Res..

