# Supplementary material for: Gene Expression Profile Induced by Two Different Variants of Street Rabies Virus in Mice
Source: Viruses. 2022 Mar 27;14(4):692. doi: 10.3390/v14040692 (PMC9031335; doi:10.3390/v14040692)
Supplement: Supplementary file 1 [file viruses-14-00692-s001.zip › PDF Supplementary Data Reviewed.pdf]

Supplementary tables and figures

S1. Averaged weigh with standard deviation (SD) of C57/BL6 mice infected with wild-type rabies virus strain variant 2 (V2) and variant 3 (V3)(n=8). Animals were observed during a 30-day period. Lethality rate was 100% for V2 and 62.5% for V3.

Observation Days	V2 (grams ± SD)	V3 (grams ± SD)
01	17,4 ± 0,97	18,0 ± 0,67
02	17,67 ± 0,5	17,8 ± 0,92
03	17,67 ± 0,7	18,3 ± 0,67
04	18,0 ± 0,5	18,3 ± 0,67
05	17,89 ± 0,78	18,0 ± 0,67
06	18,33 ± 0,7	18,1 ± 0,74
07	18,55 ± 0,73	18,0 ± 0,67
08	18,67 ± 1,41	18,2 ± 0,63
09	17,55 ± 1,67	17,7 ± 1,06
10	16,78 ± 2,28	17,2 ± 1,40
11	16,11 ± 2,37	16,6 ± 1,90
12	14,33 ± 2,83	15,9 ± 2,56
13	13,25 ± 1,67	15,11 ± 2,57
14	13,0 ± 1,22	15,75 ± 3,65
15	13,0 ± 1,41	15,0 ± 14,1
16	12,5 ± 0,71	17,0 ± 3,46
17	12,2 ± 0,32	18,25 ± 1,50
18	-	18,50 ± 1,00
19	-	18,25 ± 0,96
20	-	18,5 ± 1,0
21	-	18,75 ± 1,26
22	-	19,0 ± 0,82
23	-	19,25 ± 0,96
24	-	19,5 ± 1,00
25	-	19,75 ± 0,50
26	-	19,75 ± 1,26
27	-	19,75± 0,5
28	-	19,75 ± 0,5
29	-	20,0 ± 0,82
30	-	20,0 ± 1,63

S2. Gene Ontology (GO) biological processes terms represented by genes upregulated in mice inoculated with variant 2 (V2) compared with mice inoculated with diluent

GO_ID	All gene count	GOI gene ids	GOI gene count	GO_Name	pval	qval
GO:0002376	408	Cd74 Lbp Zbp1 H2-T23 Cd180 Tmem173 Oas3 Oas2 Psmb9 H2-Aa Tlr3 Ifitm3 C3 Lgals9 Serping1 H2-Q7 Hck Cfh Cd274 Themis2 Irgm1 Fcgr1 Irf7 Myd88 Ifi30	25	immune system process	3.33E-24	2.18E-21
GO:0045087	455	Lbp Zbp1 Cd180 Tmem173 Oas3 Oas2 Tlr3 Ifitm3 C3 Serping1 Hck Cfh Trim12a Irgm1 Fcgr1 Cybb Irf7 Irgm2 Myd88	19	innate immune response	1.59E-15	1.04E-12
GO:0051607	184	Zbp1 Tmem173 Oas3 Oas2 Apobec1 Tlr3 Ifitm3 Rtp4 Cxcl10 Trim12a Irf7	11	defense response to virus	1.19E-11	7.79E-09
GO:0009617	164	Ifi44 Usp18 Oas2 Saa3 C3 Cxcl10 Ly6a Irgm1 Fcgr1 Irgm2	10	response to bacterium	6.67E-11	4.37E-08
GO:0006955	396	Cd74 H2-T22 H2-T23 Ifi44 Oas3 Oas2 Ccl3 H2-Aa H2-Q7 Ccrl2 Cd274 Cxcl10 Myd88	13	immune response	7.23E-10	4.74E-07
GO:0032760	60	Lbp H2-T23 Ccl3 Tlr3 Lgals9 Myd88	6	positive regulation of tumor necrosis factor production	4.75E-09	3.11E-06
GO:0006954	311	Cd180 Ccl3 C3ar1 Tlr3 C3 Hck Ccrl2 Cxcl10 Themis2 Cybb Myd88	11	inflammatory response	4.97E-09	3.25E-06
GO:0032481	18	Tmem173 Tlr3 Irf7 Myd88	4	positive regulation of type I interferon production	8.24E-09	5.4E-06
GO:0035458	38	Tmem173 Tlr3 Irgm1 Igtp Irgm2	5	cellular response to interferon-beta	9.58E-09	6.28E-06
GO:0019886	9	Cd74 H2-Aa Ifi30	3	antigen processing and presentation of exogenous peptide antigen via MHC class II	3.2E-08	2.09E-05
GO:0071222	184	Lbp Cd180 Plscr2 Cd274 Cxcl10 Tspo Gbp10 Irgm2	8	cellular response to lipopolysaccharide	6.23E-08	4.08E-05
GO:0060100	12	Lbp C3 Fcgr1	3	positive regulation of phagocytosis, engulfment	1.24E-07	8.15E-05

GO:0071346	100	Ccl3 Tlr3 Gbp7 H2-Q7 Gbp10 Irgm1	6	cellular response to interferon-gamma	1.73E-07	0.000113
GO:0016064	13	Cd74 Irf7 Myd88	3	immunoglobulin mediated immune response	1.79E-07	0.000117
GO:0050830	108	Lbp H2-T23 Gbp7 Hck Gbp10 Myd88	6	defense response to Gram-positive bacterium	2.93E-07	0.000192
GO:0060337	15	Oas2 Ifitm3 Myd88	3	type I interferon signaling pathway	3.4E-07	0.000223
GO:0009615	79	Oas3 Oas2 Tlr3 Ifitm3 Myd88	5	response to virus	8.44E-07	0.000553
GO:0032722	22	Lbp Tlr3 Lgals9	3	positive regulation of chemokine production	1.78E-06	0.001167
GO:0090023	24	Cd74 Lbp C3ar1	3	positive regulation of neutrophil chemotaxis	2.57E-06	0.001685
GO:0006952	156	Cd74 Tlr3 Cxcl10 Irgm1 Igtp Irgm2	6	defense response	3.49E-06	0.002286
GO:0032757	26	Lbp Tlr3 Myd88	3	positive regulation of interleukin-8 production	3.6E-06	0.002356
GO:0051603	59	Psmb9 Casp8 Ctss Ctsc	4	proteolysis involved in cellular protein catabolic process	4.22E-06	0.002765
GO:0002476	28	H2-T22 H2-T23 H2-Q7	3	antigen processing and presentation of endogenous peptide antigen via MHC class Ib	4.9E-06	0.003207
GO:0042832	29	Gbp7 Gbp10 Irgm2	3	defense response to protozoan	5.66E-06	0.003708
GO:0034341	30	H2-Aa Ifitm3 Irgm2	3	response to interferon-gamma	6.51E-06	0.004266
GO:0032496	176	Lbp Dcn Lgals9 Vcam1 Hpgd Myd88	6	response to lipopolysaccharide	7.72E-06	0.00506
GO:0031663	33	Lbp Ccl3 Myd88	3	lipopolysaccharide-mediated signaling pathway	9.63E-06	0.00631
GO:0006935	130	Ccl3 C3ar1 Lgals9 Ccrl2 Cxcl10	5	chemotaxis	1.53E-05	0.010052

GO:0001916	40	H2-T22 H2-T23 H2-Q7	3	positive regulation of T cell mediated cytotoxicity	2.11E-05	0.013789
GO:0050918	45	Ccl3 Saa3 Angpt1	3	positive chemotaxis	3.38E-05	0.022135
GO:0060326	92	Saa3 Ccrl2 Vcam1 Cxcl10	4	cell chemotaxis	3.73E-05	0.02444

S3. Gene Ontology (GO) molecular function terms represented by genes upregulated in mice inoculated with variant 2 (V2) compared with mice inoculated with diluent

GO_ID	All gene count	GOI gene ids	GOI gene count	GO_Name	Pval	qval
GO:0042605	36	H2-T22 H2-T23 H2-Aa H2-Q7	4	peptide antigen binding	3.42E-07	6.84E-05
GO:0003924	299	Gbp7 Mx1 Rhoj Gbp10 Gbp11 Irgm1 Igtp Irgm2	8	GTPase activity	3.64E-06	0.000727
GO:0005525	384	Gbp7 Mx1 Rhoj Gvin1 Gbp10 Gbp11 Irgm1 Igtp Irgm2	9	GTP binding	3.7E-06	0.000739
GO:0005102	388	Lbp H2-T22 H2-T23 Lgals9 Angpt1 H2-Q7 Hck Msn Myd88	9	signaling receptor binding	4.05E-06	0.00081
GO:0005518	62	Srgn Dcn Ctss Tgfbi	4	collagen binding	5.4E-06	0.001081
GO:0004197	76	Usp18 Casp8 Ctss Ctsc	4	cysteine-type endopeptidase activity	1.48E-05	0.002951
GO:0003725	77	Oas3 Oas2 Tlr3 Msn	4	double-stranded RNA binding	1.57E-05	0.003145
GO:0050839	64	Vcam1 Tgfbi Msn	3	cell adhesion molecule binding	0.000136	0.027172
GO:0005515	4387	Cd74 Zbp1 Cd180 Usp18 Tor3a Tmem173 Srgn Lgals3bp Ccl3 Apobec1 H2-Aa Xdh Casp8 Tlr3 Plscr2 C3 Lgals9 Ube2l6 Hck Lcp2 Cfh Rtp4 A530064D06Rik Cd274 Rhoj Pld4 Themis2 Ly6a Cybb Irf7 Msn Myd88	32	protein binding	0.000145	0.028902

S4. Gene Ontology (GO) cellular component terms represented by genes upregulated in mice inoculated with variant 2 (V2) compared with mice inoculated with diluent

GO_ID	ALL gene count	GOI gene ids	GOI gene count	GO Name	Pval	qval
GO:0009986	605	Cd74 Lbp H2-T23 Tlr3 Ifitm3 H2-Q7 Rtp4 Ctss Vcam1 Cd274 Cd59a Ly6c2 Msn	13	cell surface	1.5E-07	1.91E-05
GO:0005615	1592	Lbp Cd180 Srgn Dcn Lgals3bp Ccl3 Saa3 Xdh C3 Apod Angpt1 Serping1 Cfh Cp Ctss Vcam1 Cxcl10 Tgfbi Ctsc Hpgd Scgb3a1	21	extracellular space	2.78E-07	3.54E-05
GO:0005764	350	Cd74 Grn H2-Aa Ifitm3 Hck Ctss Ctsc Irgm1 Ifi30	9	lysosome	1.62E-06	0.000205
GO:0009897	451	Cd74 H2-Aa Ccrl2 Vcam1 Cd274 Cxcl10 Cd59a Ly6a Fcgr1	9	external side of plasma membrane	1.51E-05	0.001913
GO:0030670	38	H2-T23 H2-Q7 Irgm1	3	phagocytic vesicle membrane	1.71E-05	0.002173
GO:0005576	1683	Lbp Srgn Dcn Lgals3bp Ccl3 Saa3 Xdh C3 Lgals9 Apod Angpt1 Serping1 Cfh Cp Cxcl10 Tgfbi Scgb3a1 Ifi30	18	extracellular region	3.84E-05	0.00488
GO:0062023	244	Vwa5a Dcn Lgals9 Serping1 Tgfbi Ctsc	6	collagen-containing extracellular matrix	6.3E-05	0.007995
GO:0005623	108	Ccl3 C3ar1 Cp Cxcl10	4	cell	8.04E-05	0.010207
GO:0045335	69	Pld4 Irgm1 Cybb	3	phagocytic vesicle	0.000182	0.023116
GO:0031225	136	Vcam1 Cd59a Ly6a Ly6c2	4	anchored component of membrane	0.000237	0.030143
GO:0005902	74	Angpt1 Vcam1 Msn	3	microvillus	0.000239	0.030293

S5. Gene Ontology (GO) biological processes terms represented by genes downregulated in mice inoculated with variant 2 (V2) compared with mice inoculated with diluent

GO_ID	ALL gene count	GOI gene ids	GOI gene count	GO Name	pval	qval
GO:0050909	85	Vmn1r126 Vmn1r131 Vmn1r132 Vmn1r135 Vmn1r142 Gm8453 Gm5728 Vmn1r100 Vmn1r111 Vmn1r112 Vmn1r114 Vmn1r118 Gm4214 Gm4216	14	sensory perception of taste	2.68E-28	2.94E-27
GO:0007186	2091	Gm4201 Vmn1r155 Vmn1r126 Vmn1r130 Vmn1r131 Vmn1r132 Vmn1r135 Gm5725 Gm10666 Vmn1r142 Gm8653 Gm8453 Gm10665 Vmn1r151 Vmn1r152 Gm16442 Vmn1r95 Gm5728 Vmn1r100 Vmn1r104 Gm5726 Vmn1r111 Vmn1r112 Vmn1r114 Vmn1r117 Vmn1r118 Gm4214 Gm4216	28	G-protein coupled receptor signaling pathway	1.28E-25	1.41E-24
GO:0007165	2514	Gm4201 Vmn1r155 Vmn1r126 Vmn1r130 Vmn1r131 Vmn1r132 Vmn1r135 Gm5725 Gm10666 Vmn1r142 Gm8653 Gm8453 Gm10665 Vmn1r151 Vmn1r152 Gm16442 Vmn1r95 Gm5728 Vmn1r100 Vmn1r104 Gm5726 Vmn1r111 Vmn1r112 Vmn1r114 Vmn1r117 Vmn1r118 Hivep2 Gm4214 Gm4216	29	signal transduction	4.46E-25	4.91E-24
GO:0019236	199	Gm4201 Vmn1r155 Vmn1r130 Gm5725 Gm10666 Gm8653 Gm10665 Vmn1r151 Vmn1r152 Gm16442 Vmn1r95 Vmn1r104 Gm5726 Vmn1r117	14	response to pheromone	1.84E-22	2.02E-21
GO:0050896	1318	Vmn1r126 Vmn1r131 Vmn1r132 Vmn1r135 Vmn1r142 Gm8453 Gm5728 Vmn1r100 Vmn1r111 Vmn1r112 Vmn1r114 Vmn1r118 Gm4214 Gm4216	14	response to stimulus	2.42E-10	2.66E-09

S6. Gene Ontology (GO) molecular function terms represented by genes downregulated in mice inoculated with variant 2 (V2) compared with mice inoculated with diluent

GO_ID	ALL gene count	GOI.gene_ids	GOI gene count	GO Name	pval	qval
GO:0016503	231	Gm4201 Vmn1r155 Vmn1r126 Vmn1r130 Vmn1r131 Vmn1r132 Vmn1r135 Gm5725 Gm10666 Vmn1r142 Gm8653 Gm8453 Gm10665 Vmn1r151 Vmn1r152 Gm16442 Vmn1r95 Gm5728 Vmn1r100 Vmn1r104 Gm5726 Vmn1r111 Vmn1r112 Vmn1r114 Vmn1r117 Vmn1r118 Gm4214 Gm4216	28	pheromone receptor activity	6.39E-54	5.75E-53
GO:0004930	1931	Gm4201 Vmn1r155 Vmn1r126 Vmn1r130 Vmn1r131 Vmn1r132 Vmn1r135 Gm5725 Gm10666 Vmn1r142 Gm8653 Gm8453 Gm10665 Vmn1r151 Vmn1r152 Gm16442 Vmn1r95 Gm5728 Vmn1r100 Vmn1r104 Gm5726 Vmn1r111 Vmn1r112 Vmn1r114 Vmn1r117 Vmn1r118 Gm4214 Gm4216	28	G-protein coupled receptor activity	1.3E-26	1.17E-25
GO:0003674	4349	Gm4201 Vmn1r155 Vmn1r126 Vmn1r130 Vmn1r131 Vmn1r132 Vmn1r135 Gm5725 Gm10666 Vmn1r142 Gm8653 Gm8453 Gm10665 Vmn1r151 Vmn1r152 Gm16442 Vmn1r95 Gm5728 Vmn1r100 Vmn1r104 Gm5726 Vmn1r111 Vmn1r112 Vmn1r114 Vmn1r117 Vmn1r118 Gm2012 Gm4214 Gm4216	29	molecular_function	4.94E-18	4.45E-17

S7. Gene Ontology (GO) cellular component terms represented by genes downregulated in mice inoculated with variant 2 (V2) compared with mice inoculated with diluent

GO ID	ALL gene count	GOI gene ids	GOI gene count	GO Name	pval	qval

GO:0005575	3260	Gm4201 Vmn1r155 Vmn1r126 Vmn1r130 Vmn1r131 Vmn1r132 Vmn1r135 Gm5725 Gm10666 Vmn1r142 Gm8653 Gm8453 Gm10665 Vmn1r151 Vmn1r152 Gm16442 Vmn1r95 Gm5728 Vmn1r100 Vmn1r104 Gm5726 Vmn1r111 Vmn1r112 Vmn1r114 Vmn1r117 Vmn1r118 Gm4214 Gm4216	28	cellular_component	4.24E-20	3.39E-19
GO:0016021	6665	Gm4201 Vmn1r155 Vmn1r126 Vmn1r130 Vmn1r131 Vmn1r132 Vmn1r135 Gm5725 Gm10666 Vmn1r142 Gm8653 Gm8453 Gm10665 Vmn1r151 Vmn1r152 Gm16442 Vmn1r95 Gm5728 Vmn1r100 Vmn1r104 Gm5726 Vmn1r111 Vmn1r112 Vmn1r114 Vmn1r117 Vmn1r118 Gm4214 Gm4216	28	integral component of membrane	2.07E-11	1.65E-10
GO:0016020	8607	Gm4201 Vmn1r155 Vmn1r126 Vmn1r130 Vmn1r131 Vmn1r132 Vmn1r135 Gm5725 Gm10666 Vmn1r142 Gm8653 Gm8453 Gm10665 Vmn1r151 Vmn1r152 Gm16442 Vmn1r95 Gm5728 Vmn1r100 Vmn1r104 Gm5726 Vmn1r111 Vmn1r112 Vmn1r114 Vmn1r117 Vmn1r118 Gm4214 Gm4216	28	membrane	2.04E-08	1.63E-07
GO:0005886	5141	Gm4201 Vmn1r155 Vmn1r130 Gm5725 Gm10666 Gm8653 Gm10665 Vmn1r151 Vmn1r152 Gm16442 Vmn1r95 Vmn1r104 Gm5726 Vmn1r117	14	plasma membrane	0.005834	0.046675

S8. KEGG pathways represented by genes upregulated in mice inoculated with variant 2 (V2) compared with mice inoculated with diluent

**Pathway**	**p.val**	**FDR q.val**	**GOI.ids**	**Entrez.ids**	**External.ids**	**goi.count**	**All genes in pathway count**
mmu05168 Herpes simplex infection	4.05e-16	3.81E-14	Myd88, Irf7, H2-Q7, Casp8, Tlr3, Daxx, C3, H2-Aa, Oas2, Oas3, H2-T22, H2-T23, Cd74	110558, 12266, 12370, 13163, 142980, 14960, 15018, 15039, 15040, 15051, 16149, 17874, 246727, 246728, 54123	Myd88, Irf7, H2-Q7, Casp8, Tlr3, Daxx, C3, H2-Aa, Oas2, Oas3, H2-T22, H2-T23, Cd74	15	215
mmu04612 Antigen processing and presentation	3.66e-12	3.44E-10	Ctss, H2-Q7, Ifi30, H2-Aa, H2-T22, H2-T23, Cd74	110558, 13040, 14960, 15018, 15039, 15040, 15051, 16149, 65972	Ctss, H2-Q7, Ifi30, H2-Aa, H2-T22, H2-T23, Cd74	9	91
mmu04145 Phagosome	1.93e-10	1.81E-08	Fcgr1, Cybb, Ctss, H2-Q7, C3, H2-Aa, H2-T22, H2-T23	110558, 12266, 13040, 13058, 14129, 14960, 15018, 15039, 15040, 15051	Fcgr1, Cybb, Ctss, H2-Q7, C3, H2-Aa, H2-T22, H2-T23	10	181
mmu05167 Kaposi's sarcoma-associated herpesvirus infection	1.33e-09	1.25E-07	Irf7, H2-Q7, Hck, Casp8, Tlr3, C3, H2-T22, H2-T23	110558, 12266, 12370, 142980, 15018, 15039,	Irf7, H2-Q7, Hck, Casp8, Tlr3, C3, H2-T22, H2-T23	10	217

				15040, 15051, 15162, 54123			
mmu05416 Viral myocarditis	2.71e-09	2.55E-07	H2-Q7, Casp8, H2-Aa, H2-T22, H2-T23	110558, 12370, 14960, 15018, 15039, 15040, 15051	H2-Q7, Casp8, H2-Aa, H2-T22, H2-T23	7	88
mmu04620 Toll-like receptor signaling pathway	6.957e-09	6.54E-07	Myd88, Irf7, Cxcl10, Casp8, Tlr3, Ccl3, Lbp	12370, 142980, 15945, 16803, 17874, 20302, 54123	Myd88, Irf7, Cxcl10, Casp8, Tlr3, Ccl3, Lbp	7	99
mmu05330 Allograft rejection	7.535e-09	7.08E-07	H2-Q7, H2-Aa, H2-T22, H2-T23	110558, 14960, 15018, 15039, 15040, 15051	H2-Q7, H2-Aa, H2-T22, H2-T23	6	64
mmu05332 Graft-versus-host disease	8.416e-09	7.91E-07	H2-Q7, H2-Aa, H2-T22, H2-T23	110558, 14960, 15018, 15039, 15040, 15051	H2-Q7, H2-Aa, H2-T22, H2-T23	6	65
mmu04940 Type I diabetes mellitus	1.426e-08	1.34E-06	H2-Q7, H2-Aa, H2-T22, H2-T23	110558, 14960, 15018, 15039, 15040, 15051	H2-Q7, H2-Aa, H2-T22, H2-T23	6	70
mmu04621 NOD-like receptor signaling pathway	2.840e-08	2.67E-06	Myd88, Cybb, Irf7, Casp8, Gbp7, Oas2, Oas3, Tmem173	12370, 13058, 17874, 229900, 246727, 246728, 54123, 72512	Myd88, Cybb, Irf7, Casp8, Gbp7, Oas2, Oas3, Tmem173	8	168
mmu04514 Cell adhesion molecules (CAMs)	2.990e-08	2.81E-06	Cd274, Vcam1, H2-Q7, H2-Aa, H2-T22, H2-T23	110558, 14960, 15018, 15039, 15040, 15051, 22329, 60533	Cd274, Vcam1, H2-Q7, H2-Aa, H2-T22, H2-T23	8	169
mmu05320 Autoimmune thyroid disease	3.347e-08	3.15E-06	H2-Q7, H2-Aa, H2-T22, H2-T23	110558, 14960, 15018, 15039, 15040, 15051	H2-Q7, H2-Aa, H2-T22, H2-T23	6	79
mmu05152 Tuberculosis	4.682e-08	4.4E-06	Myd88, Fcgr1, Ctss, Casp8, C3, H2-Aa, Lbp, Cd74	12266, 12370, 13040, 14129, 14960, 16149, 16803, 17874	Myd88, Fcgr1, Ctss, Casp8, C3, H2-Aa, Lbp, Cd74	8	178
mmu05150 Staphylococcus aureus infection	1.063e-07	9.99E-06	Fcgr1, Cfh, C3, H2-Aa, C3ar1	12266, 12267, 12628, 14129, 14960	Fcgr1, Cfh, C3, H2-Aa, C3ar1	5	56
mmu05140 Leishmaniasis	3.152e-07	2.96E-05	Myd88, Fcgr1, Cybb, C3, H2-Aa	12266, 13058, 14129, 14960, 17874	Myd88, Fcgr1, Cybb, C3, H2-Aa	5	67
mmu05164 Influenza A	4.301e-07	4.04E-05	Myd88, Irf7, Cxcl10, Tlr3, H2-Aa, Oas2, Oas3	142980, 14960, 15945, 17874, 246727, 246728, 54123	Myd88, Irf7, Cxcl10, Tlr3, H2-Aa, Oas2, Oas3	7	168

mmu05203 Viral carcinogenesis	4.305e-07	4.05E-05	Irf7, H2-Q7, Casp8, C3, H2-T22, H2-T23	110558, 12266, 12370, 15018, 15039, 15040, 15051, 54123	Irf7, H2-Q7, Casp8, C3, H2-T22, H2-T23	8	231
mmu04610 Complement and coagulation cascades	1.599e-06	0.00015	Cd59a, Serping1, Cfh, C3, C3ar1	12258, 12266, 12267, 12509, 12628	Cd59a, Serping1, Cfh, C3, C3ar1	5	88
mmu05166 HTLV-I infection	2.254e-06	0.000212	Tspo, Vcam1, H2-Q7, H2-Aa, H2-T22, H2-T23	110558, 12257, 14960, 15018, 15039, 15040, 15051, 22329	Tspo, Vcam1, H2-Q7, H2-Aa, H2-T22, H2-T23	8	282
mmu05145 Toxoplasmosis	5.289e-06	0.000497	Igtp, Myd88, Irgm1, Casp8, H2-Aa, LT629149.4	12370, 14960, 15944, 16145, 17874	Igtp, Myd88, Irgm1, Casp8, H2-Aa, LT629149.4	5	108
mmu04623 Cytosolic DNA-sensing pathway	6.327e-06	0.000595	Irf7, Cxcl10, Zbp1, Tmem173	15945, 54123, 58203, 72512	Irf7, Cxcl10, Zbp1, Tmem173	4	64
mmu04622 RIG-I-like receptor signaling pathway	8.540e-06	0.000803	Irf7, Cxcl10, Casp8, Tmem173	12370, 15945, 54123, 72512	Irf7, Cxcl10, Casp8, Tmem173	4	68
mmu04210 Apoptosis	1.983e-05	0.001864	Ctsc, Parp3, Ctss, Casp8, Daxx	12370, 13032, 13040, 13163, 235587	Ctsc, Parp3, Ctss, Casp8, Daxx	5	136
mmu05162 Measles	1.983e-05	0.001864	Myd88, Msn, Irf7, Oas2, Oas3	17698, 17874, 246727, 246728, 54123	Myd88, Msn, Irf7, Oas2, Oas3	5	136
mmu05142 Chagas disease (American trypanosomiasis)	5.840e-05	0.005489	Myd88, Casp8, C3, Ccl3	12266, 12370, 17874, 20302	Myd88, Casp8, C3, Ccl3	4	101
mmu04217 Necroptosis	8.662e-05	0.008142	Cybb, Parp3, Casp8, Tlr3, Zbp1	12370, 13058, 142980, 235587, 58203	Cybb, Parp3, Casp8, Tlr3, Zbp1	5	177
mmu05134 Legionellosis	9.242e-05	0.008688	Myd88, Casp8, C3	12266, 12370, 17874	Myd88, Casp8, C3	3	58
mmu04218 Cellular senescence	0.000114	0.010693	H2-Q7, H2-T22, H2-T23	110558, 15018, 15039, 15040, 15051	H2-Q7, H2-T22, H2-T23	5	186
mmu05160 Hepatitis C	0.000221	0.020827	Irf7, Tlr3, Oas2, Oas3	142980, 246727, 246728, 54123	Irf7, Tlr3, Oas2, Oas3	4	134
mmu05133 Pertussis	0.000264	0.024847	Myd88, Serping1, C3	12258, 12266, 17874	Myd88, Serping1, C3	3	76
mmu05169 Epstein-Barr virus infection	0.000282	0.026562	H2-Q7, H2-T22, H2-T23	110558, 15018, 15039, 15040, 15051	H2-Q7, H2-T22, H2-T23	5	220
mmu05132 Salmonella infection	0.000292	0.027454	Myd88, Ccl3, Lbp	16803, 17874, 20302	Myd88, Ccl3, Lbp	3	78

mmu05161 Hepatitis B	0.000299	0.028146	Myd88, Irf7, Casp8, Tlr3	12370, 142980, 17874, 54123	Myd88, Irf7, Casp8, Tlr3	4	143
mmu05323 Rheumatoid arthritis	0.000370	0.034825	Angpt1, Ccl3, H2-Aa	11600, 14960, 20302	Angpt1, Ccl3, H2-Aa	3	83

S9. Heatmap of normalised expression levels (Robust Multichip Average RMA) normalised for all differentially expressed probes. Sample numbers and treatment groups are shown by coloured bars at head of figure. Raw data used to generate the figure are available as part of supplemental table S10.

Separated file

S10. Combined significant DEGs in raw data used to generate the figure S9

Separated file
