# Supplementary material for: Gene Expression Profile Induced by Two Different Variants of Street Rabies Virus in Mice
Source: Viruses. 2022 Mar 27;14(4):692. doi: 10.3390/v14040692 (PMC9031335; doi:10.3390/v14040692)
Supplement: Supplementary file 1 [file viruses-14-00692-s001.zip › S10 combined.significant.table.pdf]

PROBEID	ENTREZID	SYMBOL	GENENAME	logFC	AveExpr	t	P.Value	adj.P.Val
17213213	12370	Casp8	caspase 8	1,069118	5,447792	4,232604	0,00024	0,042001
17225169	10030374(	Snora75	small nucle	-1,16028	4,741718	-4,75222	5,97E-05	0,034375
17227696	12628	Cfh	complemer	1,052275	7,029563	4,848927	4,61E-05	0,034375
17228563	30935	Tor3a	torsin famil	1,871276	6,121992	4,262254	0,000222	0,040552
17231754	15273	Hivep2	human imr	-1,1028	5,556762	-4,06011	0,000379	0,049468
17236800	13179	Dcn	decorin	1,306738	7,826494	4,437108	0,000139	0,038162
17241409	19073	Srgn	serglycin	1,00669	6,079287	4,621968	8,47E-05	0,03517
17248380	16822	Lcp2	lymphocyt	1,213547	6,042437	4,464992	0,000129	0,038162
17249028	68662	Scgb3a1	secretoglob	1,28489	5,268578	4,312193	0,000194	0,038633
17249980	16145	Igtp	interferon g	2,974146	5,550341	4,125868	0,000319	0,046566
17249990	54396	Irgm2	immunity-r	2,684462	5,613745	4,144019	0,000304	0,045646
17254153	327978	Slfn5	schlafen 5	1,360386	4,975748	4,327127	0,000186	0,038162
17256959	14824	Grn	granulin	1,108191	7,957545	4,92072	3,8E-05	0,033567
17262202	15944	Irgm1	immunity-r	2,441011	4,275307	4,054578	0,000385	0,049891
17266520	16859	Lgals9	lectin, gala	1,705668	6,820865	4,10158	0,00034	0,046831
17266967	20302	Ccl3	chemokine	3,264857	5,23184	4,071182	0,000368	0,048965
17272785	19039	Lgals3bp	lectin, gala	2,502764	6,810053	4,445933	0,000136	0,038162
17276386	80837	Rhoj	ras homolo	1,407991	6,582188	4,745139	6,09E-05	0,034375
17279404	104759	Pld4	phospholip	1,381248	6,098356	4,121458	0,000322	0,046742
17287827	21810	Tgfbi	transformi	1,034342	6,000241	4,395757	0,000155	0,038162
17289527	17079	Cd180	CD180 anti	1,248632	4,701029	4,125702	0,000319	0,046566
17313654	12257	Tspo	translocato	1,197963	5,109653	4,740267	6,17E-05	0,034375
17315743	18414	Osmr	oncostatin	1,79603	5,956613	4,805899	5,17E-05	0,034375
17316780	11600	Angpt1	angiopoieti	1,349273	4,943732	5,217675	1,71E-05	0,025647
17318083	110454	Ly6a	lymphocyt	1,493597	9,481318	4,413195	0,000148	0,038162
17318100	10004154(	Ly6c2	lymphocyt	2,490514	5,407911	6,152595	1,44E-06	0,010555
17321768	668218	Bin2	bridging in	1,021542	5,678484	4,150496	0,000298	0,045333
17324446	67775	Rtp4	receptor tr	2,38226	5,595447	4,304087	0,000198	0,039222
17329759	11815	Apod	apolipopro	1,204386	10,26663	4,623937	8,43E-05	0,03517
17332531	17857	Mx1	MX dynami	2,594316	3,220527	4,271327	0,000216	0,040469
17336213	13163	Daxx	Fas death d	1,028005	6,523138	4,713536	6,63E-05	0,034375
17337120	110558	H2-Q9	histocompa	1,944598	7,198823	4,20467	0,000258	0,042791
17337133	15018	H2-Q7	histocompa	1,256673	4,652003	4,469592	0,000127	0,038162
17343789	16912	Psmb9	proteasom	1,292373	4,180072	4,409672	0,00015	0,038162
17343813	14960	H2-Aa	histocompa	1,235358	3,750365	4,558614	0,0001	0,03517
17344568	15040	H2-T23	histocompa	2,36769	5,578945	4,35605	0,000173	0,038162
17344593	15039	H2-T22	histocompa	2,046997	6,450271	4,364582	0,000169	0,038162
17345775	328830	A530064D(	RIKEN cDN/	1,827119	2,242878	4,572044	9,68E-05	0,03517
17346528	12266	C3	complemer	2,088814	4,763462	4,467455	0,000128	0,038162
17347163	22436	Xdh	xanthine de	1,73111	5,274755	4,463551	0,000129	0,038162
17350982	16149	Cd74	CD74 antig	1,147766	5,893017	4,08594	0,000354	0,047856
17353663	72512	Sting1	stimulator	1,208278	4,893706	5,454852	9,09E-06	0,022726
17357810	17476	Mpeg1	macrophag	1,931391	5,921186	5,119839	2,23E-05	0,027569
17358544	60533	Cd274	CD274 anti	2,653008	5,394054	4,090571	0,00035	0,047427
17362973	68774	Ms4a6d	membrane-	2,397453	4,323593	4,248898	0,00023	0,0411
17372604	56791	Ube2l6	ubiquitin-c	1,59175	5,785736	4,115425	0,000328	0,046742
17373825	12509	Cd59a	CD59a anti	1,179815	6,629352	4,372449	0,000165	0,038162
17377870	15162	Hck	hemopoiet	1,023282	5,733819	4,076961	0,000363	0,048459
17378827	16803	Lbp	lipopolysac	1,169438	5,844373	4,352838	0,000174	0,038162
17378958	71878	Fam83d	family with	1,004083	5,051379	4,56459	9,88E-05	0,03517
17381589	16425	Itih2	inter-alpha	1,079089	5,296318	5,481566	8,47E-06	0,022726

17387517	12258	Serping1	serine (or c	2,263992	6,032748	5,904537	2,76E-06	0,010555
17395079	58203	Zbp1	Z-DNA bind	2,309874	4,886709	4,129689	0,000315	0,046529
17396260	12870	Cp	ceruloplasr	1,568504	6,405712	6,160144	1,41E-06	0,010555
17400375	13040	Ctss	cathepsin S	1,372844	7,651575	4,661771	7,61E-05	0,03517
17403224	229900	Gbp7	guanylate b	2,759224	5,652197	4,069597	0,00037	0,049013
17408024	14129	Fcgr1	Fc receptor	1,498704	5,455737	4,581649	9,44E-05	0,03517
17409649	22329	Vcam1	vascular cel	1,128548	6,732631	4,480295	0,000124	0,038162
17411147	99899	Ifi44	interferon-i	3,539649	6,313094	4,190766	0,000268	0,043268
17421394	627585	Gm13034	SWI/SNF re	1,025732	5,259023	5,045909	2,72E-05	0,029545
17424608	12517	Cd72	CD72 antig	1,442695	4,546023	6,075125	1,76E-06	0,010555
17430906	230787	Themis2	thymocyte	1,159981	4,964451	4,371977	0,000165	0,038162
17449718	15945	Cxcl10	chemokine	3,549577	4,821178	4,392533	0,000157	0,038162
17450501	626578	Gbp10	guanylate-b	2,665263	5,721583	4,495504	0,000119	0,038096
17450515	634650	Gbp11	guanylate b	2,041019	3,365434	4,37412	0,000164	0,038162
17452054	246728	Oas2	2'-5' oligoa	1,886607	4,760257	4,592252	9,17E-05	0,03517
17452070	246727	Oas3	2'-5' oligoa	1,025718	4,70279	4,208727	0,000256	0,04267
17462437	24110	Usp18	ubiquitin s	3,599722	6,078524	4,091617	0,000349	0,047427
17470580	11810	Apobec1	apolipopro	1,386461	4,62178	4,343216	0,000179	0,038162
17470616	12267	C3ar1	complemer	1,046532	4,61767	4,775453	5,61E-05	0,034375
17474726	10031247	Vmn1r95	vomeronas	-1,65672	4,35928	-4,65364	7,78E-05	0,03517
17474728	10004353	Vmn1r100	vomeronas	-1,53963	4,350289	-4,2912	0,000205	0,039751
17474730	667135	Vmn1r104	vomeronas	-1,53802	3,993094	-4,28812	0,000207	0,039787
17474736	667530	Vmn1r155	vomeronas	-1,45887	4,339436	-4,20267	0,00026	0,042791
17474738	10004356	Vmn1r112	vomeronas	-1,43682	4,005896	-4,33882	0,000181	0,038162
17474759	10004359	Vmn1r130	vomeronas	-1,44659	4,402684	-4,37393	0,000165	0,038162
17474761	10004360	Vmn1r132	vomeronas	-1,38983	3,995863	-4,26602	0,000219	0,040469
17474765	667404	Vmn1r135	vomeronas	-1,43099	4,587049	-4,17083	0,000283	0,044245
17474774	10004359	Vmn1r130	vomeronas	-1,44659	4,402684	-4,37393	0,000165	0,038162
17474789	667504	Vmn1r152	vomeronas	-1,57199	4,008018	-4,56026	9,99E-05	0,03517
17474795	667530	Vmn1r155	vomeronas	-1,45887	4,339436	-4,20267	0,00026	0,042791
17474801	667404	Vmn1r135	vomeronas	-1,43099	4,587049	-4,17083	0,000283	0,044245
17474807	10004359	Vmn1r130	vomeronas	-1,44659	4,402684	-4,37393	0,000165	0,038162
17480018	13032	Ctsc	cathepsin C	1,727475	6,098192	5,696987	4,77E-06	0,015933
17487509	620401	Vmn1r240	vomeronas	-1,47763	4,14045	-4,21838	0,000249	0,04259
17487511	10004303	Vmn1r250	vomeronas	-1,57409	4,262153	-4,91128	3,9E-05	0,033567
17487515	667472	Vmn1r251	vomeronas	-1,37397	4,165545	-4,8185	5E-05	0,034375
17487517	667094	Vmn1r252	vomeronas	-1,5499	4,483197	-4,89339	4,09E-05	0,033809
17487525	10004296	Vmn1r-ps7	vomeronas	-1,32848	4,3138	-4,32829	0,000186	0,038162
17487527	435949	Vmn1r244	vomeronas	-1,23266	4,257556	-4,69313	7E-05	0,034375
17487531	10004305	Gm10665	predicted g	-1,47194	4,104166	-4,518	0,000112	0,03741
17487535	435946	Vmn1r245	vomeronas	-1,29634	4,360292	-4,25524	0,000226	0,040955
17487537	10004306	Vmn1r256	vomeronas	-1,48147	4,971241	-5,15745	2,01E-05	0,026855
17487543	667292	Vmn1r111	vomeronas	-1,53225	4,675028	-4,47235	0,000126	0,038162
17487550	10004299	Vmn1r114	vomeronas	-1,57591	4,28833	-4,96297	3,39E-05	0,033184
17487554	667262	Vmn1r117	vomeronas	-1,26236	4,108082	-4,51406	0,000113	0,03741
17487556	667259	Vmn1r118	vomeronas	-1,56868	4,460687	-4,95654	3,45E-05	0,033184
17487568	10004301	Vmn1r126	vomeronas	-1,49556	4,283987	-4,69259	7,01E-05	0,034375
17487582	10004301	Vmn1r131	vomeronas	-1,47967	4,686373	-4,35686	0,000172	0,038162
17487584	10004307	Vmn1r258	vomeronas	-1,49931	4,274339	-4,85455	4,54E-05	0,034375
17487586	10004308	Vmn1r259	vomeronas	-1,51324	4,047991	-4,59711	9,05E-05	0,03517
17487588	435940	Vmn1r249	vomeronas	-1,2963	4,348379	-4,39119	0,000157	0,038162
17487596	10004301	Vmn1r131	vomeronas	-1,47967	4,686373	-4,35686	0,000172	0,038162

17487603	620401	Vmn1r240	vomeronas	-1,47763	4,14045	-4,21838	0,000249	0,04259
17487605	10004303	Vmn1r250	vomeronas	-1,57409	4,262153	-4,91128	3,9E-05	0,033567
17487607	667464	Vmn1r142	vomeronas	-1,44746	4,697718	-4,24484	0,000232	0,0411
17487609	667472	Vmn1r251	vomeronas	-1,37397	4,165545	-4,8185	5E-05	0,034375
17487611	667094	Vmn1r252	vomeronas	-1,5499	4,483197	-4,89339	4,09E-05	0,033809
17487615	10004296	Vmn1r-ps7	vomeronas	-1,32848	4,3138	-4,32829	0,000186	0,038162
17487617	435949	Vmn1r244	vomeronas	-1,23266	4,257556	-4,69313	7E-05	0,034375
17487621	10004305	Gm10665	predicted g	-1,47194	4,104166	-4,518	0,000112	0,03741
17487623	435947	Vmn1r151	vomeronas	-1,36104	4,274677	-4,64551	7,95E-05	0,03517
17487625	435946	Vmn1r245	vomeronas	-1,29634	4,360292	-4,25524	0,000226	0,040955
17487627	10004306	Vmn1r256	vomeronas	-1,48147	4,971241	-5,15745	2,01E-05	0,026855
17487645	10004307	Vmn1r258	vomeronas	-1,49931	4,274339	-4,85455	4,54E-05	0,034375
17487647	10004308	Vmn1r259	vomeronas	-1,51324	4,047991	-4,59711	9,05E-05	0,03517
17487649	435940	Vmn1r249	vomeronas	-1,2963	4,348379	-4,39119	0,000157	0,038162
17487657	10004301	Vmn1r131	vomeronas	-1,47967	4,686373	-4,35686	0,000172	0,038162
17491193	20210	Saa3	serum amyl	1,357044	4,079367	4,315282	0,000192	0,038633
17494353	76681	Trim12a	tripartite m	1,871508	5,218571	5,373067	1,13E-05	0,025647
17494651	668108	Gvin-ps3	GTPase, ver	1,540302	6,236728	4,568788	9,77E-05	0,03517
17494656	74558	Gvin1	GTPase, ver	1,07967	5,170443	4,189619	0,000269	0,043268
17494664	668128	Gvin-ps5	GTPase, ver	1,453451	6,202583	4,35256	0,000174	0,038162
17497718	66141	Ifitm3	interferon i	2,222975	7,262467	4,251693	0,000228	0,0411
17497813	54123	Irf7	interferon r	2,995763	5,700588	4,17624	0,000279	0,044245
17501250	15446	Hpgd	hydroxypro	1,044786	6,16359	4,119483	0,000324	0,046742
17509101	142980	Tlr3	toll-like rec	1,518493	7,778675	4,114462	0,000328	0,046742
17510136	65972	Ifi30	interferon g	1,569063	5,729214	5,218116	1,71E-05	0,025647
17516194	67776	Vwa5a	von Willebr	1,177907	6,337184	4,134427	0,000311	0,046152
17520326	18828	Plscr2	phospholip	1,889062	4,150472	4,442775	0,000137	0,038162
17530703	235587	Parp3	poly (ADP-r	1,328359	5,253153	5,275026	1,47E-05	0,025647
17531705	54199	Ccrl2	chemokine	1,078725	4,378008	4,107585	0,000334	0,046795
17532137	17874	Myd88	myeloid dif	1,178154	5,675487	4,612405	8,69E-05	0,03517
17532509	102680	Slc6a20a	solute carri	1,033612	8,193955	4,388543	0,000158	0,038162
17536364	17698	Msn	moesin	1,057142	7,543026	4,41866	0,000146	0,038162
17540154	13058	Cybb	cytochrom	2,049965	4,624303	4,610812	8,73E-05	0,03517
17540736	10003903	Gm2012	predicted g	-1,50745	4,373806	-4,24905	0,00023	0,0411
17548436	10086175	Gm21190	predicted g	-1,62819	4,45919	-4,14935	0,000299	0,045333

B	Change	Experiment	Control_5 1	Variant3_5 10	Control_5 11	Control_10 12
0,571958	Up	Variant2_1	5,2	5,2	5,1	5,6
1,813871	Down	Variant2_1	5	4,7	5,5	5,7
2,045411	Up	Variant2_1	7,1	6,8	7	6,7
0,642523	Up	Variant2_1	5,4	5,6	5,9	5,8
0,162769	Down	Variant2_1	6,1	5,9	5,8	6,2
1,059678	Up	Variant2_1	9,1	8,2	8	7
1,501945	Up	Variant2_1	5,6	6,1	5,8	5,8
1,126329	Up	Variant2_1	5,7	6,2	5,4	5,9
0,7615	Up	Variant2_1	5,3	5	4,6	4,4
0,318468	Up	Variant2_1	4,4	4,4	4,5	5,3
0,361512	Up	Variant2_1	4,6	4,3	4,9	4,8
0,797107	Up	Variant2_1	4,1	4,9	4,8	4,6
2,217213	Up	Variant2_1	7,7	7,7	7,5	7,8
0,149698	Up	Variant2_1	3,8	3,6	3,8	3,5
0,260916	Up	Variant2_1	6,5	6,3	6,1	6,1
0,188963	Up	Variant2_1	4,2	4,5	4	4,6
1,08077	Up	Variant2_1	6,4	6,3	6,2	6,2
1,796904	Up	Variant2_1	6,1	6,6	6,9	5,8
0,308015	Up	Variant2_1	5,6	5,9	5,1	6,2
0,960891	Up	Variant2_1	5,7	6	6	5,7
0,318074	Up	Variant2_1	4,4	5	3,6	4,5
1,785238	Up	Variant2_1	4,9	4,8	5	4,4
1,9424	Up	Variant2_1	5,7	5,9	5,5	5,3
2,926037	Up	Variant2_1	5,1	5,1	5,6	4,4
1,002542	Up	Variant2_1	8,8	9,1	9,1	9,3
5,11588	Up	Variant2_1	4,3	5,3	4,9	4,6
0,376877	Up	Variant2_1	4,9	6	4,9	5,9
0,742179	Up	Variant2_1	4,9	5,4	4,9	4,8
1,50666	Up	Variant2_1	9,7	10,2	10,4	9,5
0,664128	Up	Variant2_1	3,2	2,5	2,2	2,5
1,721222	Up	Variant2_1	6,1	6	6,5	6
0,505533	Up	Variant2_1	6,4	6,6	7,2	6,3
1,137326	Up	Variant2_1	4,6	4,5	4	3,9
0,994126	Up	Variant2_1	4,1	3,7	3,8	3,8
1,350286	Up	Variant2_1	3,5	3,8	3,4	3,1
0,866104	Up	Variant2_1	4,7	4,8	4,8	4,6
0,886466	Up	Variant2_1	5,6	5,9	6,3	5,8
1,382428	Up	Variant2_1	2	1,8	1,9	2
1,132218	Up	Variant2_1	4	4,2	4,5	3,8
1,122885	Up	Variant2_1	4,6	5	5,1	5,1
0,223886	Up	Variant2_1	5,3	5,6	5,6	5,4
3,488798	Up	Variant2_1	4,2	4,6	4	4,7
2,692919	Up	Variant2_1	5,2	6,2	5	5,5
0,234848	Up	Variant2_1	4,7	4,8	4,8	5
0,610729	Up	Variant2_1	3,4	4	3,6	4,6
0,293717	Up	Variant2_1	5,5	5,8	5,4	5,2
0,905242	Up	Variant2_1	6,4	6,9	7,4	5,8
0,202636	Up	Variant2_1	5,4	5,7	5	5
0,85844	Up	Variant2_1	6	5,6	5,8	5,1
1,364589	Up	Variant2_1	4,3	5	5,2	4,6
3,551934	Up	Variant2_1	5,3	5,1	5,4	4,8

4,5432	Up	Variant2_1	5,5	5,5	6,1	4,9
0,327527	Up	Variant2_1	4,3	4,1	4,4	4,1
5,133196	Up	Variant2_1	6,2	6,4	6,4	5,5
1,597255	Up	Variant2_1	7	7,8	7,2	7,1
0,185213	Up	Variant2_1	4,7	5	4,9	4,7
1,40542	Up	Variant2_1	4,9	5,2	4,7	5,2
1,162919	Up	Variant2_1	6,7	6,4	6,2	6,7
0,47249	Up	Variant2_1	5,1	5,6	5,3	5,4
2,516466	Up	Variant2_1	5,3	4,8	5,7	4,3
4,937796	Up	Variant2_1	4	4,4	4	4,6
0,904116	Up	Variant2_1	4,5	4,9	4,6	4,4
0,953194	Up	Variant2_1	4,1	4	4	4
1,199291	Up	Variant2_1	5,3	5,7	5,5	5
0,909231	Up	Variant2_1	2,6	2,7	2,9	2,2
1,430802	Up	Variant2_1	4,5	4,4	4,3	4,1
0,515177	Up	Variant2_1	4,5	4,6	4,6	4,2
0,237325	Up	Variant2_1	5,4	5,3	5,1	5,4
0,835484	Up	Variant2_1	4,3	4,6	4,5	4,5
1,869498	Up	Variant2_1	4	4,4	4,1	4,3
1,577788	Down	Variant2_1	4,7	4	5,4	5,1
0,711462	Down	Variant2_1	4,7	4,2	5,3	5,2
0,704121	Down	Variant2_1	4,4	3,4	5	4,6
0,500772	Down	Variant2_1	4,7	4,1	5,4	5,1
0,824995	Down	Variant2_1	4,5	3,7	5,1	4,6
0,90877	Down	Variant2_1	4,8	4,2	5,4	5
0,651497	Down	Variant2_1	4,5	3,7	5	4,5
0,425149	Down	Variant2_1	5	4,3	5,6	5,2
0,90877	Down	Variant2_1	4,8	4,2	5,4	5
1,354229	Down	Variant2_1	4,4	3,6	5	4,7
0,500772	Down	Variant2_1	4,7	4,1	5,4	5,1
0,425149	Down	Variant2_1	5	4,3	5,6	5,2
0,90877	Down	Variant2_1	4,8	4,2	5,4	5
4,058898	Up	Variant2_1	5,9	5,8	5,7	5,5
0,53813	Down	Variant2_1	4,5	3,7	5	4,9
2,194623	Down	Variant2_1	4,8	3,9	5,3	4,9
1,972559	Down	Variant2_1	4,6	4,1	5	4,8
2,151817	Down	Variant2_1	5,2	4	5,2	5,4
0,799872	Down	Variant2_1	4,8	4	5,2	4,9
1,672363	Down	Variant2_1	4,8	4,1	5,2	5,1
1,253104	Down	Variant2_1	4,6	3,7	4,9	4,7
0,625823	Down	Variant2_1	4,8	4,3	5,2	4,9
2,782588	Down	Variant2_1	5,4	4,8	5,8	5,8
1,143924	Down	Variant2_1	5,3	4,5	5,4	5,5
2,318259	Down	Variant2_1	4,7	4	5,3	5
1,24367	Down	Variant2_1	4,6	4,1	5	4,6
2,302884	Down	Variant2_1	5,1	4,1	5,2	5,3
1,671064	Down	Variant2_1	4,7	3,9	5,4	4,9
0,868039	Down	Variant2_1	5,2	4,2	5,4	5,5
2,058878	Down	Variant2_1	4,7	3,9	5,4	4,9
1,442428	Down	Variant2_1	4,7	3,7	5	4,9
0,949996	Down	Variant2_1	4,8	4	5,1	4,9
0,868039	Down	Variant2_1	5,2	4,2	5,4	5,5

0,53813	Down	Variant2_1	4,5	3,7	5	4,9
2,194623	Down	Variant2_1	4,8	3,9	5,3	4,9
0,601073	Down	Variant2_1	5,1	4,5	5,7	5,7
1,972559	Down	Variant2_1	4,6	4,1	5	4,8
2,151817	Down	Variant2_1	5,2	4	5,2	5,4
0,799872	Down	Variant2_1	4,8	4	5,2	4,9
1,672363	Down	Variant2_1	4,8	4,1	5,2	5,1
1,253104	Down	Variant2_1	4,6	3,7	4,9	4,7
1,558317	Down	Variant2_1	5	4	5,2	5,2
0,625823	Down	Variant2_1	4,8	4,3	5,2	4,9
2,782588	Down	Variant2_1	5,4	4,8	5,8	5,8
2,058878	Down	Variant2_1	4,7	3,9	5,4	4,9
1,442428	Down	Variant2_1	4,7	3,7	5	4,9
0,949996	Down	Variant2_1	4,8	4	5,1	4,9
0,868039	Down	Variant2_1	5,2	4,2	5,4	5,5
0,768864	Up	Variant2_1	3,8	4	3,6	3,5
3,29517	Up	Variant2_1	5,5	4,8	4,9	4,7
1,374637	Up	Variant2_1	5,8	6	6	5,9
0,469767	Up	Variant2_1	4,3	5,1	4,7	4,8
0,857775	Up	Variant2_1	5,8	5,5	6	5,9
0,617383	Up	Variant2_1	6,6	6,3	6,6	6,8
0,437987	Up	Variant2_1	4,7	5,3	5	5
0,303334	Up	Variant2_1	5,9	6	6	5,8
0,291435	Up	Variant2_1	7	7,6	7,6	7,4
2,927085	Up	Variant2_1	5,4	5,5	5,5	4,9
0,33876	Up	Variant2_1	6,1	6,3	6,2	6,2
1,073222	Up	Variant2_1	3,4	3,6	3,9	3,7
3,062448	Up	Variant2_1	5	4,7	5,4	4,6
0,275142	Up	Variant2_1	3,9	4,4	4	4,2
1,479049	Up	Variant2_1	5	5,2	5,3	5,4
0,943664	Up	Variant2_1	8,7	7,7	8,3	7,6
1,015599	Up	Variant2_1	7	7,4	7,8	7,3
1,475235	Up	Variant2_10.vs.Control_10				
0,611096	Down	Variant2_10.vs.Control_10				
0,374168	Down	Variant2_10.vs.Control_10				

Control_10 13	Control_10 15	Variant3_5 16	'ariant3_5 1	'ariant3_5 1	/ariant2_5 :	:ontrol_5 2(	:ontrol_5 2:
4,6	4,8	5,7	5,6	5,8	5,4	5,6	4,2
5	5,1	4,5	4,4	3,8	4,8	5	5,3
6,4	6,2	7,3	7,3	7,5	6,9	6,9	5,7
5,7	5,7	5,8	6	6	5,5	5,8	5,7
6,2	5,5	5,1	4,9	4,8	6,1	5,1	6,1
6,6	7	8,3	8,8	8,5	7,2	7,6	6,9
5,5	5,5	6,1	6,2	6,4	5,9	6,1	5,2
5	5,6	5,9	6,2	5,9	6,2	6	5,2
4,9	5,1	5,1	5,2	5,1	5	4,7	5,2
5	4,7	5,2	5,2	5	4,9	4,8	4,4
4,7	5,1	5,6	5,6	5,8	5,1	5	4,7
4,7	4,8	4,7	4,7	4,9	4,8	4,8	4,7
7,3	7,4	7,9	8	8,1	7,7	8	7,3
3,8	3	4	3,9	3,7	3,8	4,5	2,9
6,1	6,6	6,4	6,7	6,6	6,6	6,3	6,4
4,5	4,8	4,7	4,8	5,6	4,6	5,1	4,2
5,7	6,2	6,7	6,8	6,7	6,4	6,3	5,3
6,4	6,5	6,5	6,8	6,8	6,4	6,3	6,4
5,2	5,2	6,2	6,3	6,4	5,9	5,9	4,1
5,6	5,7	6,5	6,1	6,3	5,8	5,7	5,4
4,2	4,1	4,7	4,6	4,9	4,2	4,5	3,7
4,5	5,1	4,9	4,9	5,1	5	4,8	5
5,3	5,1	5,7	6	6,1	5,3	5,9	5,2
4,3	4,6	4,9	5,3	5,3	3,9	5,1	5,3
9	8,8	9,6	9,7	9,6	9,1	9,3	8,4
5,1	3,9	5,4	5,7	5,4	4,9	5	4,6
5	4,6	6	6,1	6	5,7	5,7	4
5,1	5,1	5,3	5,4	4,9	4,9	5,3	4,7
10,2	10,3	10,3	10,5	10,5	9,8	10,3	10,1
2,9	2,5	2,7	2,4	2,8	2,7	3,7	2,7
6,1	6,3	6,6	6,5	6,3	6,5	6,3	6,6
6,6	7	7,3	7,7	7,3	6,6	6,6	7,1
4,1	4,3	4,9	4,5	4,7	4,5	4,3	4,5
3,5	3,8	4	4,4	4,2	4,2	3,9	4
3,6	3,8	4	4,1	4	3,4	4,4	3,2
5,1	4,4	5,6	5,3	5,1	5	5,8	4,5
6,1	5,9	6,5	6,4	6,4	6,2	6,4	5,4
1,7	1,9	2,5	1	1,2	1,4	2,2	2,7
3,7	4,6	5,2	5,2	4,9	4,1	4,4	3,8
4,9	4,5	5	5,3	4,9	4,4	4,8	5
5,3	6	6,3	6,2	6,1	5,2	5,7	5,3
4,1	4	5,1	4,9	5	4,6	5,2	4,2
5,1	4,4	5,8	6	6,2	5,9	5,5	3,9
4,7	4,8	5	5,2	5,2	4,8	4,9	4,5
3,4	3,2	4,2	4,4	4	4,2	3,2	3
4,8	5,3	5,5	5,2	5,5	5,3	5,4	5,3
6,3	6,7	7,1	7,4	7,5	6,1	6,5	6,6
5,5	5,6	5,6	5,8	5,9	5,6	5,6	5
5,2	5,3	6,8	5,6	6,3	5,7	5,4	5,1
5,2	4,1	5,1	4,6	5,1	5,1	5,3	4,8
4,6	4,7	6	5,9	5,8	4,7	5,7	4,6

4,4	5,1	6,1	6	6,3	5,5	6	5,9
4,4	4,6	4,5	4,8	4,3	3,7	4,6	4,3
6	5,3	6,7	6,6	6,8	5,9	6,4	5,8
6,9	6,7	7,6	7,7	8,1	7,7	7,2	5,5
4,7	4,8	5,4	5,6	5,6	5,3	5,3	3,5
4,9	4,6	5	5,3	4,9	5,1	5,2	5,1
6,2	6,4	6,7	6,8	6,9	6,7	6,7	5,6
5,3	5,5	5,8	6	6	5,3	5,4	5,6
5,2	4,6	5,7	5,2	5,4	5	5	5,1
4,5	4	4,3	4,5	4,1	4,1	4,3	4
4,7	4,5	5	4,7	5,1	4,7	4,9	4,1
3,7	4,2	3,6	4,5	4,3	4	4,2	3,7
4,9	5,2	5,7	5	5,6	5	5,4	5,3
3,3	3,4	2,4	2,8	3,4	2,7	2,6	2,5
4,1	4,3	4,6	4	4,3	4,5	4,5	4,5
4,3	4,2	4,6	4,8	4,7	4,5	4,4	4,7
5,1	5,2	5,9	5,5	5,6	5,4	5,5	4,7
4,2	4,1	4,8	4,4	4,7	4,2	4,5	3,8
4,2	4	4,8	4,8	4,7	4,3	4,5	3,7
6,3	4,8	4,1	3,3	3,6	4,4	4,7	7,1
6,4	4,7	3,8	3,4	3,5	4,3	4,2	7,1
5,7	4,4	3,6	3,6	3,2	4,2	4,3	6,7
6,1	4,7	4,1	3,3	3,3	4,2	4,4	7,1
5,8	4,4	3,6	3,7	3,2	4	3,7	6,5
6,1	4,8	4,1	3,5	3,6	4,2	4,6	7,1
6	4,1	3,5	3,4	3,3	3,6	4,1	6,4
6,4	4,9	4	3,8	3,9	4,5	4,8	7,3
6,1	4,8	4,1	3,5	3,6	4,2	4,6	7,1
5,7	4,3	3,3	3,5	3,3	4,3	4,3	6,7
6,1	4,7	4,1	3,3	3,3	4,2	4,4	7,1
6,4	4,9	4	3,8	3,9	4,5	4,8	7,3
6,1	4,8	4,1	3,5	3,6	4,2	4,6	7,1
4,9	5	6,4	6,1	6,6	5,7	6,1	4,7
6,2	4,4	3,7	3,2	3,6	4,4	4	6,7
6,2	4,6	3,7	3,7	3,8	4	4,1	6,8
5,4	4,6	3,7	3,7	3,4	4,3	4,3	6,6
6,4	5,2	3,6	4,1	3,7	4,5	4,6	7
6	4,5	3,7	3,7	3,6	3,9	4,2	6,7
5,2	4,9	3,9	3,7	3,4	4,1	4,3	6,7
6	4,3	3,4	3,4	3,5	3,8	4	6,7
6	4,7	3,8	3,6	3,6	4	4,3	6,7
6,5	5,5	4,2	4,7	4,2	5	5,1	7,4
6,6	5,3	3,7	4,4	3,5	4,6	4,9	7,2
6,2	4,6	3,7	3,7	3,8	4,1	4,1	6,8
5,4	4,4	3,7	3,8	3,4	4	4,3	6,6
6,4	5,1	3,6	4,2	3,6	4,5	4,6	6,9
6,2	4,5	3,8	3,8	3,8	4,1	4,1	6,8
6,6	5,3	4	4,4	3,5	4,6	4,9	7,2
6,1	4,5	3,8	4	3,8	3,8	4,5	6,8
6	4,4	3,4	3,1	3,5	3,8	4	6,6
6	4,4	4,1	3,7	3,6	4,3	5,1	6,7
6,6	5,3	4	4,4	3,5	4,6	4,9	7,2

6,2	4,4	3,7	3,2	3,6	4,4	4	6,7
6,2	4,6	3,7	3,7	3,8	4	4,1	6,8
6,4	4,9	4,3	3,6	3,5	4,6	4,6	7,2
5,4	4,6	3,7	3,7	3,4	4,3	4,3	6,6
6,4	5,2	3,6	4,1	3,7	4,5	4,6	7
6	4,5	3,7	3,7	3,6	3,9	4,2	6,7
5,2	4,9	3,9	3,7	3,4	4,1	4,3	6,7
6	4,3	3,4	3,4	3,5	3,8	4	6,7
5,8	4,7	3,6	3,9	3,8	4	4,2	6,7
6	4,7	3,8	3,6	3,6	4	4,3	6,7
6,5	5,5	4,2	4,7	4,2	5	5,1	7,4
6,1	4,5	3,8	4	3,8	3,8	4,5	6,8
6	4,4	3,4	3,1	3,5	3,8	4	6,6
6	4,4	4,1	3,7	3,6	4,3	5,1	6,7
6,6	5,3	4	4,4	3,5	4,6	4,9	7,2
3,9	3,6	3,5	4,2	4	4	3,3	3,8
4,8	5	5	4,9	4,6	4,9	4,8	5,3
5,7	5,9	6,1	6,2	6,4	6,1	5,6	5,5
5,1	4,8	5	5,1	5,1	5,2	5	5,1
5,8	6	6	6,2	6,3	6,1	5,5	5,6
6,4	6,5	7,2	7,3	7,2	6,8	7,1	6,5
4,8	4,8	5,2	5,2	5,3	5	4,7	4,3
5,7	5,5	6,2	6,3	6,5	6	6,4	6
7,6	7,1	7,8	7,9	7,7	7,4	7,6	6,8
5,1	4,9	5,5	5,6	5,8	5,5	5,7	5,4
6,1	6,2	6,3	6,4	6,4	6	6,2	5,3
3,5	3,3	4	4,2	4,2	3,8	3,9	4,1
5	5	4,9	5,2	4,9	5,2	4,8	5,2
4,3	4,1	4,4	4,3	4,5	3,6	4,2	3,9
5,2	5,6	5,7	5,6	5,7	5,5	5,5	5,3
7,2	7,5	8,8	8,4	8,5	7,8	8,4	7,7
6,9	7,3	7,6	7,8	7,7	7,3	7,4	8,1

ontrol_5 2	ariant3_10	ariant3_10	ariant3_10	ariant3_10	ariant2_10	Variant2_5	ariant2_5	ariant2_10
5,5	5,5	5,3	5,8	5,7	6,7	5	5,7	5,5
5	4,6	4,2	4,7	3,8	3,3	5	5,5	4,3
7,3	7,3	6,9	7,3	7,1	7,9	7,2	7	7,1
5,5	6	5,9	8,1	5,6	8,9	5,6	6	6,1
5,6	5,5	5,1	5	5,2	4,4	5,5	5,6	4,9
9,1	7,4	7,8	7,4	7,7	8,5	7,5	7,6	7,7
5,8	6,2	5,8	6,9	6,1	7,5	6,1	5,8	6,2
6,2	6,1	5,9	7,1	5,8	7,6	6,1	5,9	6
5	5,2	4,9	5,7	5	7,6	5,6	4,9	5,2
4,3	5,6	5,1	9,3	4,8	9,7	4,8	5,2	5,5
4,8	5,6	5,4	8,6	5,4	9,3	5,1	5,3	5
4,1	5,1	4,5	6,4	4,9	6,8	5,1	4,8	5
7,8	8,1	8,1	8,9	8	9,5	7,9	8	8,1
3,6	3,9	4,2	6,8	4,2	7,7	3,8	4,2	3,5
6,5	6,6	6,8	8,4	6,6	9,3	6,3	6,6	6,6
4,4	5,1	4,5	8,6	4,5	8,4	4,8	4,8	4,9
6,4	7	6,7	9,6	6,6	10,1	6,2	6,6	6,5
6,7	6,2	6,1	6,7	6,3	9,1	6,4	6,5	6,4
6	6,4	6,2	7,1	6,3	8,1	5,9	6,4	6,4
6	6	5,9	6,3	5,9	6,7	5,9	5,9	6,1
4,7	4,7	4,7	5,4	4,8	6,1	4,9	4,8	4,7
5,1	4,8	5,2	6,1	4,9	6,7	5,3	5,1	5,2
5,7	6	5,8	7,1	6	8,8	5,8	5,9	5,8
5,4	4,7	4,7	5,5	4,6	5,8	4,3	5	5,2
9	9,3	9,3	10,8	9,2	11,5	9,8	9,2	9,4
4,5	5,4	4,9	6,7	5,1	7,4	5,4	5,3	5,5
5,1	6,4	6,1	6,2	5,9	6,8	5,4	5,9	6,1
5,1	5,7	5,5	8,5	5,2	8,7	4,9	5,5	5,2
10	9,9	10,2	11,2	10,2	11,7	10,1	9,9	10,1
2,5	2,5	2,3	6,1	3,5	6,3	2,6	2,4	3,3
6,6	6,6	6,5	7,5	6,5	7,8	6,3	6,6	6,6
6,5	7,1	6,9	9	7	10,1	6,9	6,8	6,9
4,3	4,6	4,8	5,3	4,4	6,5	4,9	4,6	4
4,2	3,8	3,8	5,2	3,9	6,1	4,6	3,9	4
3,5	3,7	4	3,7	3,7	5	3,2	3,6	3,7
5,3	5,5	5	8	5,4	8,8	5,1	5,9	5,4
6,2	6,6	6,4	8,4	6,5	9,3	6,2	6,3	6
2,9	1,9	1,7	3,9	2,2	4,5	1,6	1,5	2,1
4,1	4,6	4,4	6,3	4,5	6,9	4,5	4,5	4,7
4,7	5,2	5	7,4	5,2	7,5	4,7	5	5,1
6	6	6	6,7	6,1	7,2	5,6	5,8	5,8
4,6	5,1	5,1	5,3	5,1	6,3	5	5,2	4,8
5,5	6,1	5,9	7,4	6	8,1	5,9	6,3	6,3
5,1	4,9	4,9	8,5	5	9,1	4,9	4,7	5,1
3,8	4	4	7,1	3,6	7,7	3,6	4,5	4,3
5,3	5,6	5,7	7,7	5,7	8	5,3	5,7	5,4
6,4	6,5	6,4	6,5	6,6	7,6	6,6	6,2	7,3
5,3	5,9	5,8	6,8	6	7,3	5,6	6,1	5,7
5,6	6,3	6,3	6,6	6,3	7	5,4	6,1	6,6
4,4	5,2	4,9	5,8	4,8	6,1	4,9	5,3	5,8
5,4	5,2	5,5	5,5	5,5	5,5	5,2	5	5,3

6,1	5,9	6,1	7,4	5,9	8,3	5,7	5,8	5,8
4,7	4,6	4,3	7,5	4,3	8,5	4,4	4	4,8
6,4	6,2	6,2	7,4	6,6	7,8	6,3	6,2	6,4
7,5	7,9	7,6	8,8	7,9	9,1	7,7	8,1	8,1
5,1	5,7	5,3	9	5,7	9,4	5,3	5,4	4,9
4,8	5,4	5,5	6,9	5,4	7,4	5,1	5,8	5,4
6,6	7	6,8	7,5	6,7	8,2	6,7	6,9	6,8
5,5	5,8	5,3	10,7	5,8	11	5,8	5,8	5,7
4,9	5,3	5,5	5,7	6	5,7	5,2	5,5	5,5
3,9	5	4,6	5,4	4,5	6,1	4,3	4,4	4,9
4,7	4,9	5	5,9	4,8	6,5	5	5,1	4,8
3,8	4,2	4,1	8,5	4,1	9,6	4,3	4,3	4,5
4,6	5,7	4,9	8,4	5,6	8,9	5,3	5,5	5,5
3,1	3	3,1	4,4	3,3	6,1	3,2	3,2	3
4,6	4,3	4,2	6,9	4,4	7,4	4,5	4,6	4,7
4,4	4,3	4,7	5,3	4,3	6	4,7	4,2	4,4
4,9	5,8	5,2	10,5	5,3	10,9	5,3	5,6	5,3
4,3	4,6	4,5	5,8	4,3	6,6	4,1	4,4	4,5
4,9	4,6	4,7	5,4	4,7	5,7	4,6	4,8	4,6
5,6	3,6	3,8	3,6	3,9	3,5	3,7	3,4	3,6
5,4	3,8	3,8	3,3	4	3,6	3,7	3,8	3,5
5,3	3,3	3,5	3,3	3,7	3,1	3	3,1	3,2
5,5	3,9	3,7	3,6	3,7	3,5	3,8	3,4	3,8
5,2	3,2	3,4	3,3	3,3	3,2	3,5	3,4	3,3
5,6	3,9	3,8	3,6	3,9	3,6	3,9	3,4	3,8
5,3	3,4	3,5	3	3,8	3,2	3,3	3,9	3,3
5,8	3,8	4,1	3,8	4,4	3,8	3,8	3,6	3,8
5,6	3,9	3,8	3,6	3,9	3,6	3,9	3,4	3,8
5,2	3,4	3,5	3,4	3,7	3,1	3,3	3,2	3,2
5,5	3,9	3,7	3,6	3,7	3,5	3,8	3,4	3,8
5,8	3,8	4,1	3,8	4,4	3,8	3,8	3,6	3,8
5,6	3,9	3,8	3,6	3,9	3,6	3,9	3,4	3,8
5,7	6,3	6,3	6,9	6,4	7,8	6,2	6,4	6,5
5,2	3,4	3,2	3,7	3,1	3,5	4	3,3	3,5
5,5	3,9	3,8	3,5	4	3,5	3,5	3,4	3,6
5	3,4	3,6	3,6	4	3,2	4,1	3,5	3,5
5,4	3,6	4,3	3,7	4,2	3,6	3,9	3,8	3,5
5,8	3,9	4,2	3,7	3,9	3,7	3,4	3,8	3,8
5,2	3,6	3,8	3,8	3,4	3,4	3,9	4,1	3,8
5,2	3,7	3,8	3,3	3,8	3,1	3,4	3,8	3,6
5	4,1	3,8	3,8	4,1	3,7	4,1	3,4	3,9
5,8	4,8	4,6	4,1	4,7	4	4,4	4,5	4,2
5,6	3,7	4,6	3,9	4,3	3,7	4,2	4	3,7
5,4	4	3,8	3,6	4	3,6	3,6	3,4	3,7
5	3,2	3,4	3,6	3,9	3,4	4,1	3,6	3,2
5,3	3,6	4,4	3,7	4,1	3,6	3,8	3,8	3,5
5,4	3,9	3,8	3,7	3,8	3,6	3,6	3,6	3,6
5,6	3,6	4,4	3,9	4,3	3,8	4,4	4	3,7
5,4	3,9	3,8	3,7	3,8	3,7	3,6	3,4	3,6
5,1	3,4	3,6	3,3	2,9	3,1	3,7	3,6	3,3
5,1	3,3	3,9	3,9	4,2	3,5	4,1	3,7	3,8
5,6	3,6	4,4	3,9	4,3	3,8	4,4	4	3,7

5,2	3,4	3,2	3,7	3,1	3,5	4	3,3	3,5
5,5	3,9	3,8	3,5	4	3,5	3,5	3,4	3,6
5,8	3,9	4,4	4,1	3,8	3,9	4,3	4	4,1
5	3,4	3,6	3,6	4	3,2	4,1	3,5	3,5
5,4	3,6	4,3	3,7	4,2	3,6	3,9	3,8	3,5
5,8	3,9	4,2	3,7	3,9	3,7	3,4	3,8	3,8
5,2	3,6	3,8	3,8	3,4	3,4	3,9	4,1	3,8
5,2	3,7	3,8	3,3	3,8	3,1	3,4	3,8	3,6
5,2	3,4	3,9	3,3	4	3,2	3,7	3,8	3,5
5	4,1	3,8	3,8	4,1	3,7	4,1	3,4	3,9
5,8	4,8	4,6	4,1	4,7	4	4,4	4,5	4,2
5,4	3,9	3,8	3,7	3,8	3,7	3,6	3,4	3,6
5,1	3,4	3,6	3,3	2,9	3,1	3,7	3,6	3,3
5,1	3,3	3,9	3,9	4,2	3,5	4,1	3,7	3,8
5,6	3,6	4,4	3,9	4,3	3,8	4,4	4	3,7
3,6	3,6	3,6	5,3	3,5	5,8	3,6	3,8	4,1
5,1	5,4	5,1	6,6	4,9	7,5	4,8	5,2	5,2
5,9	6,3	6	7,8	6,2	7,9	6	6,1	6,1
4,8	5,3	5	6,2	5,1	6,3	4,9	4,7	5,1
5,7	6,3	6	7,8	6,1	7,8	5,9	6,1	6,1
7,1	7	7	9,9	6,5	10	6,9	7,1	7
4,8	5,3	5,4	9,3	5,1	10	5,4	5,2	5,3
5,7	6,3	6,3	5,6	6,3	8,3	6,1	6,4	6,4
7,4	7,6	7,4	9,5	7,6	9,7	7,5	7,7	7,6
5,6	6,2	5,8	6,4	5,7	8,1	5,5	5,8	5,6
5,9	6,3	6,1	7,1	6,4	8,2	5,8	6,2	6,3
4,1	4,3	4,2	5,9	4	6,3	3,6	3,9	3,9
4,9	5,3	4,9	6,2	5,1	6,9	4,8	5,1	5,5
3,9	4,4	4,1	5,6	3,7	5,6	4,3	4,3	4,7
5,8	5,6	5,5	6,7	5,7	7,2	5,5	5,8	5,8
8,7	8,1	7,9	8,8	8	8,8	8,4	7,9	8,2
7,4	7,4	7,1	8	7,4	9	7,3	7,7	7,5

ariant2_10	ariant2_10	ariant2_5 3	ontrol_10	Variant3_5	Variant2_5	ariant3_10	ariant2_10	ontrol_10 9
5,7	6,4	5,6	5,2	5,4	5,1	5	6,4	5,2
5	4,1	5	5,7	4	4,9	4,9	4,2	5,2
7,3	7,9	7,1	6,5	7	6,6	7,1	7,8	6,9
5,9	8,5	5,6	5,5	5,9	5,8	5,7	8,2	5,6
5,4	4,7	5,8	6,3	6,3	5,7	6,1	5,6	6,3
7,7	8,8	7,6	7	8,6	7,4	8	8,6	7,1
6,1	6,9	6,2	5,9	5,9	5,9	5,9	7	5,9
6,1	7,1	5,8	5,7	5,9	5,6	5,9	7,3	5,9
5,3	6,9	5,2	4,9	5,2	5,1	5,4	6	5,3
5,6	9,6	5,1	5	4,8	4,7	5,1	9,5	5
5,6	9,1	5,1	4,8	5,2	4,9	5	8,9	5,1
5	6,8	5	4,3	4,6	4,6	4,6	6,4	4,9
7,9	8,9	7,9	7,6	7,8	7,7	7,6	9	7,7
4,1	7,3	3,9	3,7	4	3,8	3,9	7,3	3,9
6,6	9,2	7,1	6,3	6,4	6,6	6,3	8,9	6,9
4,9	10,2	4,8	4,6	4,1	4,2	4,6	10	3,7
6,7	9,7	6,6	5,8	6,5	5,8	6,4	9,6	6,1
6,7	7,9	6,5	5,8	6,3	6,7	6,3	7,5	5,9
6,2	7,1	6,1	5,8	5,9	5,7	5,6	7,5	6
5,8	6,9	5,9	5,6	5,5	5,6	6,1	7,8	5,6
4,5	6,4	4,7	4,2	4,3	4,2	4,3	6,3	4,8
5,2	6,4	4,6	4,8	5,1	4,8	4,6	6,2	4,9
5,8	7,7	6	5,5	5,5	5,7	5,7	7,6	5,5
4,7	6,1	5,1	3,8	4,5	5,3	4,8	6	4
9,7	11,1	9,6	9	9,4	8,9	8,9	11,3	9,4
5,9	7,9	5,6	4,5	5,2	5	5	8,7	4,8
5,8	6,2	5,8	5,3	6,1	5,3	5,4	6,3	5,4
5,8	8,4	5,4	4,6	5,2	5,2	4,9	8,5	5
10,5	11,5	10,3	9,5	9,7	9,9	10,1	11,4	9,8
3	6,8	2,5	2,3	2,6	2,6	3,2	6,5	2,6
6,6	7,7	6,2	6	6,5	6,2	6,1	7,3	6,4
7	9,4	7	6,5	6,8	6,6	6,9	9,4	6,7
5	5,6	4,5	4,6	4,6	4,8	4,8	5,8	3,6
4,3	5,5	4	3,8	3,9	3,8	3,9	5,5	4
3,6	4,9	3,2	2,7	3,5	3,6	3,6	5,5	3,4
5,6	8,1	5,4	5,4	5,5	5,2	4,8	8,3	4,9
6,3	8,8	5,8	5,2	6	5,8	6,2	8,7	5,8
3,1	3,7	1,8	1,2	2,2	2	2,1	4,6	2,1
4,6	6,9	4,5	4,3	4,9	3,8	4,3	8,2	4,3
6	7,5	4,3	4,7	4,8	4,6	5,1	7,3	5,4
5,6	7,3	5,9	5,3	5,5	5,3	5,7	7,3	5,6
5	5,8	5	4,4	5,1	5,1	4,9	5,7	4,5
6	7,6	5,8	5,6	6	5,4	5,5	8	5,6
5,3	9	4,8	4,8	4,6	4,7	4,7	8,7	4,7
4,5	7	3,7	3,6	3,7	4,3	3,9	7,2	3,9
6	7,3	6	5,6	5,6	5,2	5,8	7,8	5,7
6,9	7,6	6,7	5,5	5,6	6,4	6,5	6,8	6,2
5,6	6,7	5,3	5,6	5,6	5,3	5,5	6,9	5,4
5,8	6,5	4,8	5,4	5,3	5,5	5,6	6,7	5,7
5,4	6	4,6	5	4,7	5,1	4,8	5,6	4,8
5,8	6,3	4,9	4,9	5,3	5	5,3	6	4,5

6,3	7,9	5,7	5,6	5,4	5,7	6	8,6	5,5
4,8	7,6	4,5	4,4	4,5	4,5	4,6	7,8	4,5
6,4	7,8	6	5,6	6,4	6,4	6,1	7,8	5,9
7,7	8,7	7,6	7,4	7,9	7,2	7,7	8,8	7,4
5,8	9,1	5,1	5,1	5,3	5	5	9	5
5,5	7,3	5,5	5,2	5,3	5,4	5,3	7,1	5,4
6,8	8	6,8	6,1	6,4	6	6,6	7,8	6,6
6,4	10,6	5,9	5,2	5,9	5,6	5,9	10,7	5,5
5,7	6	5,8	4,4	4,9	5,3	4,9	5,5	4,8
5	6,2	4,2	3,9	4,1	4,7	4,1	6,3	4,2
5	6,3	4,5	5	4,8	4,9	4,7	6,4	4,7
5,2	10	4	4,3	4	3,7	4,2	9,2	4,4
5,6	8,9	5,4	4,8	4,9	4,9	5,1	9,1	4,8
3,6	6,5	2,6	3,5	3,2	3,2	3,3	6,4	2,9
5,1	6,4	4,4	4,5	4,8	4,6	4,7	6,8	3,8
4,6	5,9	4,8	4,6	4,7	4,5	4,7	6,2	4,7
6,1	10,8	5,7	4,8	5,4	5,3	5,8	10,6	5
4,6	6,5	4,3	4,3	4,3	4,1	4,3	6,3	4,4
5	5,2	4,9	4	4,8	4,3	4,2	5,8	4,5
4	2,9	4,1	5,4	4,2	4,8	4	4,3	4,9
4	3,4	4,1	5,3	4,1	4,8	4	4,3	4,8
3,7	2,4	3,7	5	3,8	4,6	3,5	4	4,5
4	3,3	4	5,4	4,3	4,7	3,9	4,3	4,9
3,5	3,1	4	4,9	3,9	4,4	3,5	4	4,5
3,9	3,3	4,1	5,4	4,4	4,7	3,9	4,4	4,9
3,2	3,1	3,8	4,6	3,8	4,6	3,6	3,9	4,5
4,1	3,5	4,6	5,6	4,5	4,7	4,1	4,7	5,1
3,9	3,3	4,1	5,4	4,4	4,7	3,9	4,4	4,9
3,7	2,4	3,7	5	3,9	4,5	3,5	4	4,6
4	3,3	4	5,4	4,3	4,7	3,9	4,3	4,9
4,1	3,5	4,6	5,6	4,5	4,7	4,1	4,7	5,1
3,9	3,3	4,1	5,4	4,4	4,7	3,9	4,4	4,9
5,9	7,6	6,1	5,8	5,9	5,5	5,9	7,6	5,6
3,8	3,2	3,9	5,1	4	4,6	3,9	3,9	4,7
3,8	3,5	4,1	5,3	3,9	4,6	3,8	3,5	4,9
3,4	3,4	4,1	5,1	4	4,5	3,8	4	4,4
4,1	3,7	4,5	5,2	4,2	4,7	4,2	4,4	4,9
3,8	3,5	4,3	5,3	3,4	4,5	4	4,2	4,9
4,2	3,4	4	4,8	4,1	4,4	3,8	3,9	4,7
3,6	3,3	3,9	5,1	4	4,3	3,7	3,8	4,6
3,9	3,6	4,3	5,2	3,9	4,9	4	4,3	5
4,5	4	4,7	5,8	4,4	5,2	4,7	4,9	5,4
4,3	3,7	4,6	5,3	4,3	5	4,6	4,6	5
3,8	3,5	4,1	5,3	3,8	4,6	3,9	3,5	4,9
3,3	3,4	4,2	4,8	3,7	4,5	3,8	4	4,4
4	3,6	4,5	5,1	4,2	4,8	4,2	4,3	4,9
3,9	3,5	4,1	5,3	3,8	4,7	3,9	3,5	4,8
4,4	3,9	4,6	5,4	4,4	5	4,7	4,6	5
3,9	3,5	4,1	5,3	3,8	4,7	3,9	3,3	4,7
3,6	3,3	4	5	3,8	4,5	3,8	3,9	4,5
3,7	3,5	4,3	5,2	4	4,8	4,2	4,1	4,6
4,4	3,9	4,6	5,4	4,4	5	4,7	4,6	5

3,8	3,2	3,9	5,1	4	4,6	3,9	3,9	4,7
3,8	3,5	4,1	5,3	3,9	4,6	3,8	3,5	4,9
4,3	3,9	4,5	5,7	4,6	5,4	4,4	4,4	5,2
3,4	3,4	4,1	5,1	4	4,5	3,8	4	4,4
4,1	3,7	4,5	5,2	4,2	4,7	4,2	4,4	4,9
3,8	3,5	4,3	5,3	3,4	4,5	4	4,2	4,9
4,2	3,4	4	4,8	4,1	4,4	3,8	3,9	4,7
3,6	3,3	3,9	5,1	4	4,3	3,7	3,8	4,6
3,9	3,7	4,3	4,9	4,1	4,5	4	4	4,7
3,9	3,6	4,3	5,2	3,9	4,9	4	4,3	5
4,5	4	4,7	5,8	4,4	5,2	4,7	4,9	5,4
3,9	3,5	4,1	5,3	3,8	4,7	3,9	3,3	4,7
3,6	3,3	4	5	3,8	4,5	3,8	3,9	4,5
3,7	3,5	4,3	5,2	4	4,8	4,2	4,1	4,6
4,4	3,9	4,6	5,4	4,4	5	4,7	4,6	5
4,5	6,1	3,9	4,1	4,3	4	3,6	5,7	4,2
5,6	7,2	4,9	4,4	4,5	5	4,5	7,1	4,4
6,3	8,3	6,3	5,3	5,8	6,1	5,8	7,9	5,9
5,2	6,5	5,2	4,6	5	5,2	5	6,8	5,1
6,3	8,3	6,3	5,3	5,8	6	5,9	7,8	5,9
7,3	9,8	7,2	6,5	6,9	6,5	7	10	6,8
5,8	9,5	5,2	5,1	5,3	5,3	5,1	9,5	5,2
6	6,7	6,3	5,7	5,9	5,9	6,1	6,5	6
7,7	9,8	7,6	7,4	7,7	7,4	7,5	9,6	7,4
5,7	6,9	5,3	5,3	5,3	5,8	5,4	7,2	5,5
6,2	7,9	6,3	5,9	6,1	5,6	6,2	7,7	6,1
3,8	6,8	4,1	3,4	3,5	3,4	4	6,2	3,6
5,3	6,8	5,1	5	4,9	4,9	5	6,8	4,9
4,4	6	4,3	3,9	4,1	4,1	4,2	5,6	4,3
5,6	7,2	5,5	5,3	5,5	5,4	5,3	6,8	5,2
8,2	9,3	7,9	8	7,9	8	8,4	8,8	7,8
7,6	8,4	7,4	7	7,1	7,9	7,1	8,5	7,2
