# Supplementary figures and images for: Gene Expression Profile Induced by Two Different Variants of Street Rabies Virus in Mice
Source: Viruses. 2022 Mar 27;14(4):692. doi: 10.3390/v14040692 (PMC9031335; doi:10.3390/v14040692)

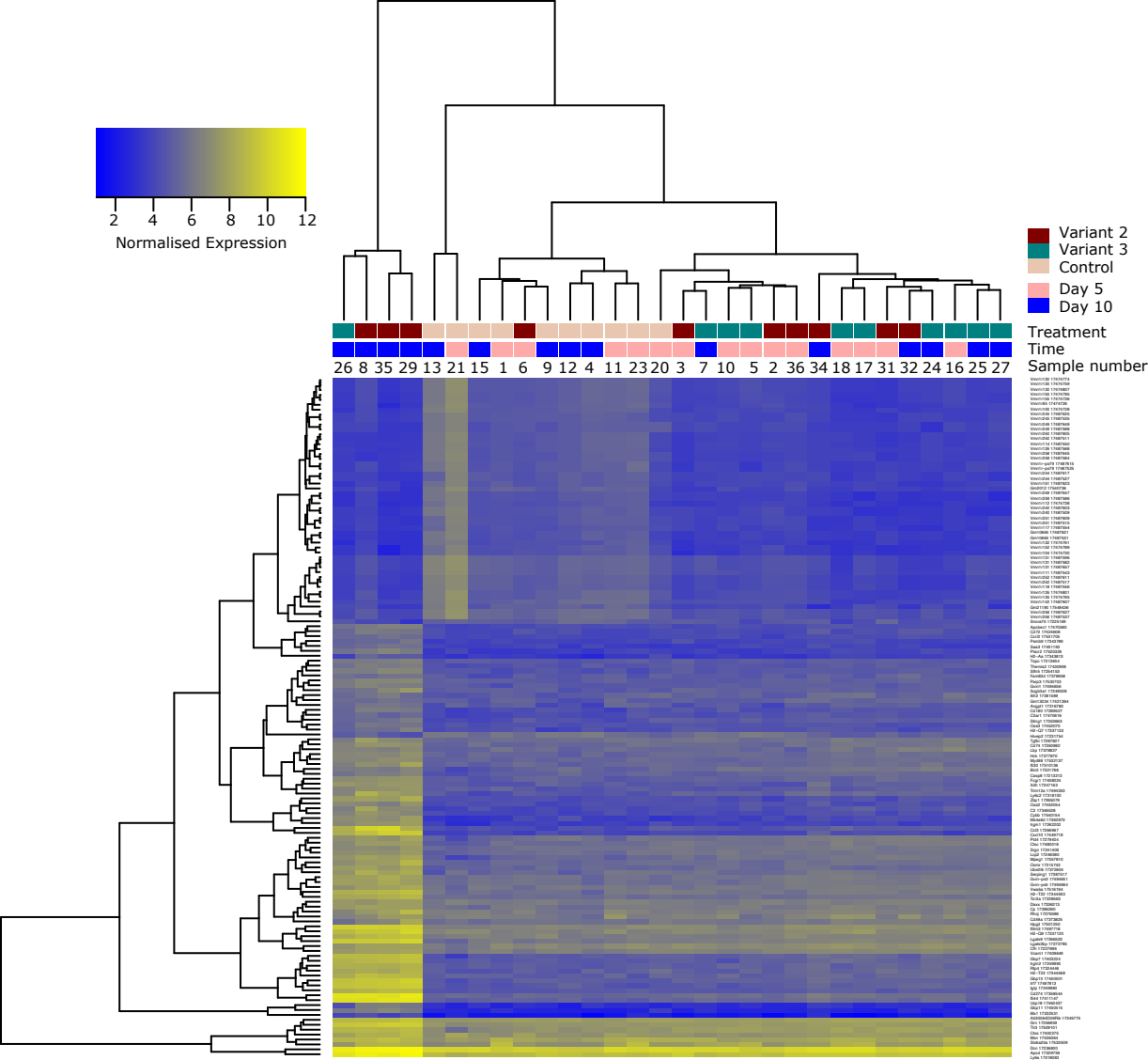

Supplement: Supplementary file 1 [file viruses-14-00692-s001.zip › S9 heatmap.pdf]
